# The Janus-Faced Role of Lipid Droplets in Aging: Insights from the Cellular Perspective

**DOI:** 10.3390/biom13060912

**Published:** 2023-05-30

**Authors:** Nikolaus Bresgen, Melanie Kovacs, Angelika Lahnsteiner, Thomas Klaus Felder, Mark Rinnerthaler

**Affiliations:** 1 Department of Biosciences and Medical Biology, Paris-Lodron University Salzburg, 5020 Salzburg, Austria; nikolaus.bresgen@plus.ac.at (N.B.);; 2 Department of Laboratory Medicine, Paracelsus Medical University, 5020 Salzburg, Austria

**Keywords:** LDs, autophagy, mitochondria, protein aggregates, lipid peroxides, misfolded proteins, mTOR, IIS, lifespan, aging

## Abstract

It is widely accepted that nine hallmarks—including mitochondrial dysfunction, epigenetic alterations, and loss of proteostasis—exist that describe the cellular aging process. Adding to this, a well-described cell organelle in the metabolic context, namely, lipid droplets, also accumulates with increasing age, which can be regarded as a further aging-associated process. Independently of their essential role as fat stores, lipid droplets are also able to control cell integrity by mitigating lipotoxic and proteotoxic insults. As we will show in this review, numerous longevity interventions (such as mTOR inhibition) also lead to strong accumulation of lipid droplets in *Saccharomyces cerevisiae*, *Caenorhabditis elegans*, *Drosophila melanogaster*, and mammalian cells, just to name a few examples. In mammals, due to the variety of different cell types and tissues, the role of lipid droplets during the aging process is much more complex. Using selected diseases associated with aging, such as Alzheimer’s disease, Parkinson’s disease, type II diabetes, and cardiovascular disease, we show that lipid droplets are “Janus”-faced. In an early phase of the disease, lipid droplets mitigate the toxicity of lipid peroxidation and protein aggregates, but in a later phase of the disease, a strong accumulation of lipid droplets can cause problems for cells and tissues.

## 1. Introduction

**Figure 1:**
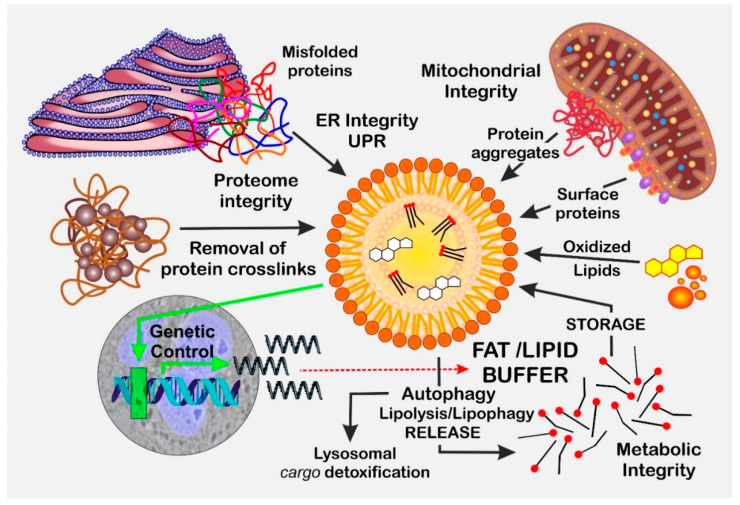
LDs as a cellular “buffer organelle”. LDs serve as an intermediate cytosolic lipid buffer and assist the cell in detoxifying lipids, misfolded proteins, and protein aggregates present in the cytosol, ER, and mitochondria. Furthermore, LDs are also involved in adaption to cellular stress by modulating transcriptional control.

Lipid droplets (LDs) are evolutionary conserved structures that were mentioned for the first time by Van Leeuwenhoek in 1674, but their reassessment as autonomous organelles with important key roles in lipid and energy metabolism occurred many years later [1,2]. LDs originate from the endoplasmic reticulum (ER). In the first step, neutral lipids are synthesized at the ER and are redirected into the bilayer, leading to an aggregation of the highly motile lipids. Morphologically, the accumulation of neutral lipids in the ER bilayer resembles a lens-like structure. Growth in this lens initiates bilayer deformation and the budding-off of LDs to the cytoplasm [3]. Due to this special mode of formation, LDs are surrounded by a lipid monolayer and are filled with neutral lipids, especially triacyclglycerols (TAGs) and sterols. Therefore, LDs are mainly considered fat-storage organelles with high relevance to lipid-metabolism homeostasis. However, in recent years, evidence has accumulated that LDs are also capable of mediating cytoprotective properties by either acting as a “buffer” for toxic lipids [4,5,6] or serving the cellular clearance of damaged and misfolded proteins [7,8,9,10,11] (Figure 1). There is also growing evidence that LDs are involved in the binding and detoxification of xenobiotics; however, this will not be discussed in detail within this review, which focuses on the aspect of aging. By studying protein composition in LDs in different organisms such as bacteria, plants, insects, yeast, and mammals, hundreds of different LD-surface-associated proteins have been identified. Although the LD proteome shows qualitative and quantitative variations among different cell types, a typical mammalian LD contains 100–150 different proteins [12]. Surface proteins are important for regulating LD homeostasis and enable the specific contract with other cell organelles. Major LD-associated proteins in mammals belong to the PAT protein family, also known as perilipin 1–5 (PLIN1-5) [13,14], adipocyte differentiation-related protein (ADRP) [15], and tail-interacting protein of 47 kDa (TIP47) [14,15]. Several different LD-resident proteins contributing to lipid biogenesis and degradation, as well as membrane trafficking and signaling, have been well reviewed [16]. Furthermore, it is well established that LDs form defined contacts with several other cellular organelles such as the ER, peroxisomes, lysosomes, and mitochondria (reviewed in [17]). Intriguingly, LDs may also sequester proteins involved in genetic control such as histones [18].

**Figure 2:**
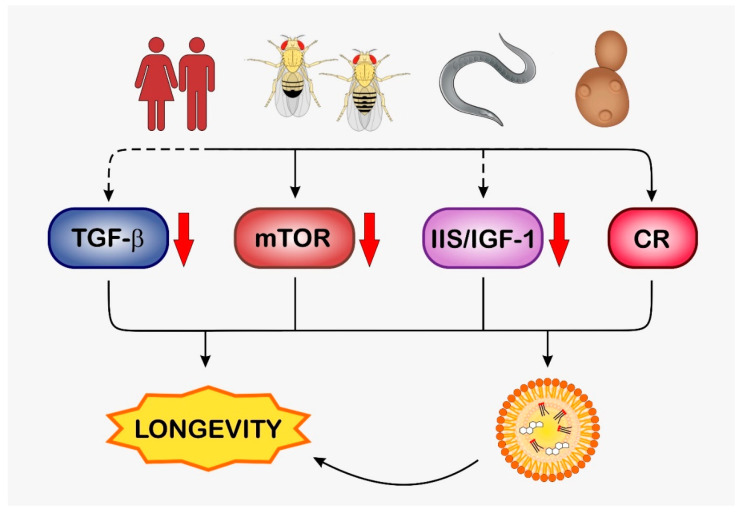
The longevity–LD interaction network. In most model organisms relevant for human aging research, inhibition of (1) TGF-β signaling, (2) mTOR signaling, (3) insulin/IGF-1 signaling and caloric restriction (CR) promotes both longevity and LD formation. Dotted lines indicate the absence of the respective pathways in yeast cells.

Here, we review the existing evidence for a distinct role of LDs in eukaryotic aging as explicitly reflected by the accumulation of LDs at terminal life periods [19,20,21]. Focusing on the “*physiologic triad*”—metabolic regulation, stress response, and aging—but also covering the evolutionary context, we decided to provide an up-to date, detailed review of a multitude of aging-related aspects of LD biology investigated in the classical biological model system (*Saccharomyces cerevisiae*, *Caenorhabditis elegans*, and *Drosophila melanogaster*) and also associated with age-related human disease. This attempt, by its nature, is complex, and discussing the fascinating, multifaceted role of lipid droplets in the multiple contexts of aging deserves an extended approach. As will be outlined, there is evidence for a Janus-faced role of LDs, their cellular accumulation counteracting stress-associated, disease-provoking forces. Thus, this is beneficial to aging, but conversely accelerates disease progression at advanced stages, which promotes the aging process. As we show in the course of this review, there is a close interplay between cellular pathways that regulate aging processes on the one hand, and on the other hand also affect the biogenesis of LDs and run like a thread through evolution (see Figure 2).

## 2. Lipid Droplets in *Saccharomyces cerevisiae*

The baker’s yeast *Saccharomyces cerevisiae* is a valuable tool for aging research, as many aging- and disease-associated pathways such as DNA repair mechanisms, lipostasis, proteostasis, oxidative stress responses, regulated cell death, nutrient signaling, autophagy, and regulation of the cell cycle are evolutionarily conserved to a high degree [22]. Based on sequence similarity, about 30% of the yeast genome is conserved in the human genome [23]. Important for aging research is the fact that when adequate and sufficient nutrients are provided, *S. cerevisiae* cells grow exponentially via asymmetric budding of daughter cells from bigger mother cells [24]. 

### 2.1. Replicative and Chronologic Lifespan 

In general, in yeast cells, two forms of aging mechanisms can be distinguished, namely, replicative and chronological aging. For both, cell death terminates the lifespan, caused by the intrinsic, mitochondrial outer membrane permeabilization (MOMP)-based activation of programmed cell death (PCD)/apoptosis emerging from increased reactive oxygen species (ROS) production and genomic instability, which provokes damage to the cellular proteome, lipidome, and organelles such as mitochondria [25]. Upon nutritional stress (i.e., exhaustion of nutrients), yeast cells stop dividing and enter a stationary phase which allows survival up to several weeks depending on strain type and culture conditions [22]. This survival period in the stationary phase is termed the chronological lifespan [26] and has to be distinguished from the yeast replicative lifespan, which is measured by the number of daughter cells that can be formed from a mother cell before it stops dividing [27]. The average lifespan in the yeast background BY4741 (the most used genetic background, generally considered as the wild type) lasts 25 generations. Aged (mother) cells are larger, and reveal a slowing down of the cell cycle and a declined protein synthesis. Each daughter cell that is formed leaves a bud scar on the mother cell surface that can be observed microscopically by calcofluor-white staining [28]. It is believed that damaged proteins and organelles (e.g., mitochondria) are specifically retained by the mother cells, which explains the “rejuvenation” of daughter cells resulting from asymmetric segregation [25,29]. Representing a mitosis-based lifespan definition, replicative aging in yeast cells mimics the limited mitotic capacity of non-transformed proliferating mammalian cells types, including undifferentiated stem cells as defined first by the *Hayflick limit* [30]. On the other hand, many phenotypical characteristic described for the chronological aging of yeast cells residing in the stationary phase share similarity with the phenotype of aged, post-mitotic cells in higher eukaryotes mainly comprising the class of terminally differentiated cell types such as cells of the central nervous system [31]. 

In some respects, several properties of *S. cerevisiae* render this fungal cell system a preferred aging model advantageous to human in vitro cell culture models. For instance, large numbers of cells can be monitored in comparatively short time periods under in vivo conditions in yeast. Of note, contrasting the well-conserved intracellular aging mechanisms common to both, yeast cells fail to display intercellular effects seen in multicellular organisms, such as inflammatory or systemic responses (e.g., regulated by hormones and/or the immune system), as well as other mechanisms involved in cell–cell communication [22]. However, it should not be overseen that, in vitro cell cultures, as for instance derived from mammalian tissues, are devoid of systemic, physiologic “cross-talks and feedback loops” if used as primary cell lines, and co-culturing with other cell types will reflect only part of the systemic complexity directing individual cell fate in vivo, in particular under the aspect of aging. Moreover, interpretation of experimental findings based on immortalized eukaryotic cell lines, self-evidently, is complicated due to the fundamentally altered growth control. 

Both replicative and chronological lifespan in yeast can be extended by caloric restriction, which can be obtained by lowering glucose availability in the culture media (e.g., from 2% to 0.5%) [32]. In the absence of caloric restriction, chronologically aged yeast cells accumulate ethanol produced by glucose fermentation [32]. It is speculated that this counteracts the expression of β-oxidation regulatory enzymes Fox1p, Fox2p, and Fox3p (peroxisomal fatty acid β-oxidation core enzymes) leading to a decline in peroxisomal oxidation of LD-derived non-esterified “free” fatty acids that are synthesized in the ER and are stored in LDs [33,34]. In turn, non-oxidized free fatty acids will accumulate in LDs under normal nutritional conditions (i.e., 2% glucose) which promotes an inhibitory feedback loop on the ER-based synthesis of triacyclglycerols (TAG) [33]. It is hypothesized that lipid dynamic remodeling of this kind can shorten lifespan in chronologically aged yeast cells grown without caloric restriction (i.e., in the presence of 2% glucose) by three different mechanisms: (i) via necrotic cell death ensuing from the peroxisomal failure to oxidize free fatty acids, (ii) apoptosis stimulated by the accumulation of diacylglycerol and free fatty acids in the ER (“*lipoapoptosis*”), or (iii) diacylglycerol initiated protein kinase C-dependent signaling [33].

This accounts for a pivotal role of lipid dynamics in yeast aging, which is further supported by the finding that LD biogenesis in yeast is elevated in the course of replicative and chronological aging as well as under stress conditions [19,35,36]. Of special relevance, Beas et al. reported that overexpression of the *BNA2* gene encoding indoleamine 2,3-dioxygenase (*BNA2* is the yeast homolog of mammalian IDO1) leads to a 40% reduction in LD accumulation during replicative aging, which identifies *BNA2* as an important regulator of LD abundance [22]. Bna2p catalyzes the first step of NAD^+^ synthesis converting tryptophan to formyl-kynurenine; hence, this finding reveals a connection between the NAD^+^/kynurenine pathway and LD formation in the course of aging. It is proposed that the glycolytic flux in aging yeast cells is directed towards neutral lipid synthesis and LD generation, but Bna2p overexpression diverts the glycolytic flux from pyruvate and acetyl-CoA to the shikimate pathway (responsible for the synthesis of the amino acids phenylalanine, tyrosine, and tryptophan) and as a result lowers LD accumulation in the aged cells. Importantly, this investigation reveals that this kind of Bna2p-mediated “metabolic rewiring” in aged yeast cells is not directly associated with longevity. Moreover, the findings indicate that LD accumulation does not cause lifespan shortening, but, conversely, exerts protection of aged cells under stress conditions, which might provide a selective growth advantage under variable environmental conditions [36]. 

### 2.2. Lipid Droplets and Stress Adaptation 

This concept is supported by another study that substantiates the role of LDs as key players in cellular stress adaption. The yeast cell growth rate declines when phosphatidylcholine biosynthesis is deficient, which changes the cellular phospholipid content and causes ER stress, alterations in ER morphology, and enhanced LD formation. In this case, an excess of phospholipids is converted to TAG by the acyltransferases Lro1p and Dga1p, which is immediately sequestered by LDs. This LD-generating process allows yeast cells to rebalance the pool of freely available phospholipids as an indispensable prerequisite for organelle morphology retrieval and cell growth [9]. Besides this pathway of ER-based regulation of lipid homeostasis yielding LD formation in yeast, ER stress arising from lipid imbalance is also at risk of activating the unfolded protein response (UPR). In most model organisms, it is shown that the UPR protects cells from the detrimental effects of proteotoxicity and is of great importance for the aging process [37]. Therefore, it is not surprising that all interventions that increase the activity of the UPR clearly extend the replicative lifespan of yeast cells [38].

The UPR provides cellular maintenance by specific handling of accumulated misfolded protein as well as facing lipid bilayer stress in the ER. Besides ER expansion, UPR signaling comprises the activity of a number of UPR-related gene products which direct the response either towards re-established homeostasis or, if not adequately facing a prolonged stress condition, participate in apoptosis onset (for a review see [39]). Essential to a successful outcome is the proper elimination of the ER stressor. A misfolded protein that initially accumulates inside the ER is translocated to the cytosol, where it is polyubiquitinylated by ubiquitin-conjugating enzymes residing at the cytosolic ER surface, the polyubiquitination serving as tag for proteasomal degradation [40]. However, lipid bilayer stress may also stimulate UPR in the ER (UPR^ER^) [41,42] which converges with the UPR triggered by the misfolded protein at the central UPR effector Ire1p (inositol-requiring enzyme 1) [43]. Interestingly, in mouse hepatocytes, ER stress stimulates Ire-1 and downstream targets such as DGAT2 (diacylglycerol-acyltransferase 2) [44], with DGAT2 (as well as DGAT1) being essential to LD biosynthesis [45]. Referring to this and findings demonstrating ROS-triggered LD biogenesis and antioxidant properties of LDs in *Drosophila* [46], Walther et al. suggested that the Ire1p/DGAT2-stimulated LD formation could antagonize phospholipid oxidation via LD-mediated ROS scavenging [47]. This also underlines the importance of LDs for the aging process as the accumulation of ROS is one of the most prominent features at the terminal lifespan [48]. 

Moreover, linking LD formation to UPR-dependent responses in yeast, it was shown that ER-derived LDs can be associated with polyubiquitinylated proteins and also can be enriched in Kar2p, an ER chaperone involved in protein folding [9]. This led to the conclusion that un-/misfolded proteins accumulating in the ER are cleared from this compartment via LD formation, the released LDs being degraded terminally in the yeast vacuole by a process resembling microautophagy, termed *microlipophagy*. It has to be emphasized that this process differs from starvation-induced macroautophagy, since it does not involve the ATG-dependent initiation of (macro)autophagosomes, but instead requires ESCRT components (endosomal sorting complexes required for transport) and the ER-stress response factor Esm1 (ER stress-induced microlipophagy protein 1) [9,10]. Both stimulation of autophagy and ESCRT components extend the chronological lifespan of yeast cells [49]. A further study also clearly links LDs with the removal of aggregates consisting of misfolded proteins. Moldavski et al. showed that so-called inclusion bodies (IBs) are functionally and spatially linked to LDs [8]. Upon stress induction, unfolded or misfolded proteins, which cannot be cleared by the quality control machinery (e.g., due to quality control system overload or failure) aggregate and form inclusion bodies. In an extensive screening approach, Moldavski and co-workers identified thirteen proteins that are crucial for an efficient and rapid IB clearance. Interestingly one of these proteins, namely, Iml2p, strongly associates with LDs via interaction with the LD-resident proteins Pet10p and Pdr16p. It should be noted that Pet10p is the yeast perilipin, which is the only perilipin discovered so far in *S. cerevisiae* [50]. This interaction especially happens during cell stress, when Iml2 is exclusively located in inclusion bodies. Under such stress conditions, a physical tethering between LDs and IBs can be monitored, the physical binding of LDs to IBs allowing aggregate clearance. Iml2 is essential to this clearance process, which is considered to be mediated by a soluble sterol derivate effusing from LDs via interaction with Iml2 [8]. These findings highlight the role of LD-dependent protein aggregate clearance during aging, which is still poorly studied considering the substantial influence of cellular aging on both protein misfolding and protein toxicity [51]. Besides Pet10p and Pdr16p, another LD-resident protein, Ubx2p, could be involved in protein homeostasis [52,53]. This UBX-domain-containing protein resides in the ER but relocates to LDs upon their formation. UBX2 deletion leads to abnormal cellular numbers of LDs of reduced size and TAG content [54]. At the same time, this protein is also involved in protein homeostasis, in that Ubx2p recruits Cdc48p and both interact to support ER-associated protein degradation [55].

### 2.3. Lipid Droplets: Guardians of Mitochondrial Integrity 

In line with these findings, our research also indicates a linkage between LD formation and the removal of un-/misfolded, potentially harmful proteins in yeast and mammalian cells. Moreover, we demonstrated that several, proteins including yeast Mmi1p and Erg6p, as well as mammalian BAX, BCL-X_L_, and TCTP, can be transferred from mitochondria to LDs via a V-shaped domain consisting of two alpha helices [35]. The V-domain shows a higher binding affinity to the LD membrane than to the outer membrane of mitochondria, which explains the directed transfer [7,35]. Among different possible contexts, this directed protein shuttling is of special relevance to the control of PCD/apoptosis onset mediated by the pro-apoptotic bcl-2 family members BAX and BAK. It has to be clearly stated that apoptosis and aging are deeply interconnected in yeast as well as in mammalian cells [56,57,58], and LDs seem to be involved in both processes. In most cells, apoptosis is increased with the dysregulation of the apoptotic program, enhancing the risk of cancer and cellular senescence [58]. Induced by a plethora of potential intrinsic cell death stimuli, BAK and BAX translocate to the mitochondrial outer membrane where they form the mitochondrial-apoptosis-induced channel (MAC), resulting in MOMP. As a consequence, the release of cytochrome C from the mitochondrial intermembrane space to the cytosol promotes apoptosome formation, caspase 9 activation, and the terminal progression of intrinsic apoptotic signaling [59,60]. Particularly under cellular stress conditions, the anti-apoptotic mammalian bcl-2 family member BCL-X_L_, as well as TCTP, also translocate to mitochondria but suppress MOMP by antagonizing BAX/BAK oligomerization [61]. In a similar way, Mmi1p, the yeast homolog of TCTP, also participates in the apoptotic machinery, with the deletion of Mmi1p leading to an extended replicative lifespan [62,63]. From this, it can be speculated that under a given stress condition both pro-and anti-apoptotic proteins locate to the outer mitochondrial membrane, continuously challenging MOMP onset. Such potentially harmful mitochondria may be specifically removed by mitophagy, a selective mode of macroautophagy [64]. 

Emphasizing its specificity for mitochondria, mitophagy in yeast depends on the activity of Uth1p which localizes to the outer mitochondrial membrane and is required for mitophagy, but not for starvation-induced bulk macroautophagy [65]. As previously stated, mitophagy is crucial to cellular maintenance under stress conditions by eliminating dysfunctional mitochondria, which is complicated by the fact that stress-induced macroautophagy/mitophagy may confer cell protection in one stress context, but conversely can contribute to cell death (i.e., autophagic cell death) under different stress conditions [66,67]. Besides BAX/BAK-mediated MAC, excessive ROS generation can lead to the formation of another mitochondrial permeability transition pore (mPT). The mPT pore complex is composed of VDAC (voltage-dependent anion channel) in the outer membrane, cyclophilin D in the matrix, and ANT (adenine-nucleotide translocator) in the inner membrane, and opening of the mPT, leading to mitochondrial swelling in many cases followed by necrotic cell death [59]. However, mPT opening may also initiate BAX/BAK-mediated MAC/MOMP; to a large degree, the outcome of this depends on cellular ATP availability comprising cell death by either necrosis or apoptosis, which also may involve enhanced autophagy/mitophagy [68]. Reminiscent of this, for yeast mutants lacking Mdm38p, a K^+^/H^+^ exchange-regulator residing in the inner mitochondrial membrane has been reported, which develops a drop of the mitochondrial membrane potential that is accompanied by mitochondrial swelling, deterioration in mitochondrial morphology, and vacuolar changes indicative of mitophagy [69]. 

LDs and mitochondrial homeostasis. It has to be emphasized that mitophagy does not necessarily need to be associated with conditions of enhanced stress, but represents an important physiological regulator of mitochondrial homeostasis. In postmitotic mammalian cells, mitophagy is crucial to the control of mitochondria numbers under normal physiologic conditions, as well as the removal of dysfunctional mitochondria in starving cells [70]. In this context, the age-dependent decline in autophagic activity seen in mammalian cells [71] deserves particular attention since it may weaken the cellular clearance from dysfunctional mitochondria. Hence, it is conceivable that additional mechanisms may support cellular maintenance in aged cells by protecting them from the onset of premature cell death via apoptosis caused by “stressed” mitochondria. The above-mentioned V-domain-based shuttling of Mmi1p, BAX, and other MOMP agonists to LDs could fulfill this task considering that LDs closely locating to mitochondria are capable of sequestering pro-apoptotic proteins, and as a result antagonize the onset of MOMP-dependent apoptosis [35]. Terminally, such potentially harmful BAX-enriched LDs will be degraded in the yeast vacuole. Indeed, in yeast cells, we demonstrated the V-domain/LD based protection from apoptosis, but, conversely, human HepG2 hepatoma cells treated with the apoptosis inducer staurosporine revealed a substantially elevated susceptibility for apoptosis upon the V-domain-mediated translocation of BAX and Bcl-X_L_ from mitochondria to LDs [35]. Explaining this, we observed the translocation of pro-apoptotic Bcl-X_S_ to the mitochondria in staurosporine-treated HepG2 cells. Opposing anti-apoptotic Bcl-X_L_ (i.e., the long isoform), Bcl-X_S_ (the short isoform) is a pro-apoptotic splice variant of Bcl-X, the Bcl-X_L_/Bcl-X_S_ ratio being defined by the cell type and cell differentiation, which are dependent (e.g., non-transformed versus tumor cells) by numerous determinants including transcription factors and cytokine signaling [72]. Importantly, we found Bcl-X_S_ to be devoid of a V-domain [23], which may explain the enhanced onset of apoptosis in staurosporine-treated HepG2 cells. Taken together, this emphasizes the dependence of V-domain/LD-based MOMP inhibition on additional regulatory elements, in particular in mammalian cells, rendering the mechanisms cell-type-specific. Ongoing research demonstrates that the V-domain-based mitochondria to LD shuttling is not restricted to the MOMP/apoptotic settings as presented above, but seem to play a more general role in cellular stress responses, as indicated by the marked protein accumulation by LDs seen during replicative aging and in the initiation of proteotoxic stress [7]. In good correspondence with this, Garcia et al. reported a substantial remodeling of the LD proteome in the presence of ER stress [10].

LDs and DNA repair. Moreover, certain yeast haploid *rad*Δ (radiation damage) deletion strains also show altered lipid storage patterns and a reduced lifespan [73]. *RAD* genes are involved in DNA repair (e.g., nucleotide/base excision repair) which is evolutionary highly conserved. In yeast, repair of double-strand breaks via homologous recombination is accomplished by the MRX complex composed of the RAD gene products Mre11p, Rad50p, and Xrs2p [73]. Deletion of one of these three genes leads to higher levels of TAGs and steryl esters, as well as characteristic changes in lipid-metabolism-associated gene expression. The down-regulated expression of lipolysis-associated genes (e.g., *TGL3*) at an augmented expression of genes involved in lipid synthesis (*LPP1*, *SLC1*), together with high TAG levels, may readily explain the observed increase in LD numbers in *rad*Δ mutants. This is accompanied by chronological lifespan shortening and pronounced mitochondrial fragmentation indicative of premature aging. However, as normally aged cells also displayed higher LD numbers, it is not clear whether the increased LD abundance simply reflects the premature aging process of *rad*Δ mutants or, vice versa, LD accumulation is causal to chronological lifespan shortening [73]. Concerning the considerations made above regarding a cytoprotective role of LD accumulation in stress adaptation, it would be interesting to study the extent to which the severity of the phenotype is altered in *rad*Δ mutants devoid of LDs. 

These findings account for a functional triad between LD abundance, mitochondrial integrity, and lifespan in yeast, which is addressed by stress conditions as well as the general aging process. Following the common view of mitochondrial dysfunction as a hallmark of aging [74], the causal relationship between mitochondrial fragmentation and chronological lifespan shortening, as seen in yeast exposed to high glucose levels [75], represents a reliable means of monitoring the aging process already at early stages [29]. Extending this, and in line with the functional triad envisaged above, LD accumulation in the same way may be considered a complementary biomarker for both premature and normal aging, as suggested by Kanagavijayan et al. [73]. With respect to this, determinants of LD synthesis such as the cellular levels of TAG and sterols are of prevalent meaning to the whole context. In yeast, two enzymes are regarded as the main actors in TAG production, Lro1p (lecithin cholesterol acyl transferase related open reading frame) and Dga1p (diacylglycerol acyltransferase 1) [76]. For sterols, the acyl-CoA:sterol acyltransferase Are1p and its paralog Are2p are the main sterol esterification tools in yeast [77]. Together these enzymes regulate the TAG:sterol balance to a ratio of 1:1 in yeast LDs [78]. We showed that the simultaneous overexpression of all Lro1p and Dga1p enzymes, as well as Are1p and Are2p (single overexpression of each enzyme), yields an extension of both the chronological and replicative lifespan of *S. cerevisiae* [19]. 

This stimulation of LD synthesis resulted in less mitochondrial fragmentation and reduced production of ROS, which normally increase during aging. Contrarily, a mutant strain devoid of LDs (*lro1*Δ, *dga1*Δ, *are1*Δ, *are2*Δ) suffers from a significantly shortened chronological lifespan and experiences a burst of ROS production already within one day of cultivation, suggesting severe mitochondrial defects [19]. According to the assumptions made above, mitochondrial functionality is an essential target for age-related cellular decline, and it seems plausible that “fitter” mitochondria with maintained integrity will be beneficial to a prolonged lifespan. 

Furthermore, mitochondria have been identified recently to assist the cytosolic proteasome in protein degradation, especially during stress conditions. Underlying this is a process termed MAGIC (mitochondria as guardian in cytosol), which mediates the import of misfolded proteins into mitochondria where protein degradation is performed by the matrix-resident protease Pim1p [79]. Yeast Pim1p is an ATP-dependent mitochondrial Lon protease required for the degradation of misfolded mitochondrial proteins, which is essential to mitochondrial function and maintenance [80]. With aging, the activity of Pim1p ceases, and *pim1*Δ yeast mutants lacking Pim1p are marked by a shortened replicative lifespan and show reduced proteasomal activity connected with an increased accumulation of oxidized and aggregated proteins in the cytosol [80]. In line with this, we also observed a significant shortening of both the replicative and chronological lifespan in *pim1*Δ cells [19]. In addition, the mitochondria of *pim1*Δ cells showed an abnormal morphology accompanied by enhanced ROS production, enlarged LDs, and a delay in the cell cycle. This premature aging phenotype of *pim1*Δ cells could be reversed partially by overexpressing Lro1p. [19] This suggests an important role of LDs in the detoxification/sequestration of the non-degraded, oxidized protein, which underlines the beneficial role of LDs in cell integrity by assisting cellular clearance from protein aggregates. 

It is noteworthy that the advantageous effects of LDs cannot be seen solely as a function of LD abundance, but also as a function of LD size and morphology. This is indicated by the observation that *pim1*Δ cells treated with oleate and olive oil showed a reduced lifespan, revealing a drop in the LD number, with the LDs themselves becoming massively enlarged. In contrast, overexpression of Lro1p/Dga1p on the *pim1*∆ restored the strains’ normal replicative lifespan but led to numerous but smaller LDs [19]. Furthermore, cells of the mutant strain *sei1*∆ (SEI, yeast seipin controls LD size, number, and morphology) show a reduced replicative lifespan but no significant differences to wild-type cells in overall neutral lipid levels. Different from the wild type, the LDs of *sei1*∆ cells are smaller and show LD clustering. Hence, LD size and distribution also obviously play an important role in the effect of LDs on lifespan in yeast [19]. In this context, it is worth mentioning that, in yeast cells, life-prolonging interventions such as caloric restriction [81], rapamycin treatment (blockage of the TOR kinase; for details see the following chapters) [82], and sirtuin inhibition [83] induce the formation of LDs [84,85,86]. In fact, in our own experiments we observed a modest 1.15–1.20-fold increase in the LD content upon treatment of BY4741 cells with 10 µM resveratrol (unpublished data).

Similar research was performed in the filamentous ascomycete *Podospora anserina* [33]. Here, deletion of the gene *PaATG24*, encoding a sorting nexin, resulted in impaired autophagy, a reduced vacuolar size, lowered growth rate, and lifespan shortening. Addition of oleic acid stimulates LD production and gives rise to an extended lifespan in wild-type as well as *PaATG24*Δ cells, revealing a restored autophagic flux and normal vacuolar phenotype. Interestingly, oleic acid treatment also diminishes ROS production in *Podospora* as result of a bypass of complex I and II of the mitochondrial electron transport chain [87]. 

Taken together, the research on LDs in yeast provides substantial evidence that LDs, apart from their well-defined role in lipid metabolism, can also serve as hitherto underrated “detoxification organelles”, which in orchestration with other processes involved in cellular maintenance, in particular the autophagic flux, serve as lifespan determinants. Such protective roles (both for proteotoxic and lipotoxic intervention) were clearly demonstrated for the model organisms discussed below. 

## 3. Lipid Droplets in *Caenorhabditis elegans*

*Caenorhabditis elegans* has proven to be one of the most important model organisms in aging research. Several milestones in this specific scientific field were achieved in this nematode. It was shown for the very first time in this worm that a mutation in a single gene (*age-1*) can extend the lifespan of a whole organism [88]. Further aging pathways that were unraveled in *C. elegans* or were studied in great detail include the insulin/IGF-1 signaling (IIS) pathway [89], TOR signaling pathway [90,91], caloric-restriction-induced signaling [92], TGF-β-signaling [93], AMPK signaling [91,94] and the HIF-1-dependent hypoxic response [95]. 

### 3.1. The C. elegans “Dauer-Larva”

To gain a better understanding of the role of LDs in the aging process of *C. elegans*, it is appropriate to provide a short overview of its lifecycle, in particular with respect to the diapause stage of the “dauer larva” resembling a suitable aging model. In *C. elegans*, two sexes can be distinguished, self-fertilizing hermaphrodites and males, each composed of an exactly defined number of somatic cells. Upon fertilization, eggs are laid. After embryonic development and hatching from these eggs, the nematodes have to pass four larval stages, each of which ends with a molt, before adulthood is reached [96]. Spectacular in this life cycle and important for aging research is the formation of a so-called dauer larva. Environmental cues including starvation-, heat-, or population-density-dependent pheromone secretion at the L2 molt phase are potential inducers of the dauer larva. In this specific phase, the worm stops eating and ceases muscular activity in the pharynx but retains full mobility. The dauer larva has a reduced intestinal lumen and specialized cuticle. As soon as the harmful environmental influences end, the larva exits the dauer stage and, after the third and fourth molts, forms an adult worm. Strikingly, this dauer stage can extend the lifespan up to 70 days, which is close to four-fold the average lifespan of an adult nematode (about 18–20 days at 20 °C) [97,98]. In the development of many longevity concepts, the C. elegans dauer larva played a significant role since all of the above-mentioned pathways (e.g., TOR, IIS, TGF-β signaling) that affect worm longevity also modulate entry of the worm into the dauer larva stage. Critically, many of the mechanisms contributing to lifespan extension have to be considered dauer-related, but also dauer-independent [99]. As we discuss in the following section, this may also apply to LDs; the dauer stage phenotype of *C. elegans* shows a close linkage to the detoxifying effects of LDs, a LD function that may also play a role in cellular maintenance in the adult worm as well as higher organisms. 

### 3.2. Detoxifying Role of Lipid Droplets 

The primary sites for fat storage in *C. elegans* are cells of the intestine and the hypodermis. In these cells, three fat deposits were identified, namely LDs, lysosome-related organelles (LROs), and undefined vesicles [100]. These sites for fat storage differ in their abundance and lipid composition. Inspection of intestinal cells by Raman scattering microscopy revealed that 18% of the cellular area is covered by LROs and 4% by LDs [101]. In contrast to yeast cells in which TAG and sterol esters are stored in LDs, there is a clear separation between LROs and LDs in *C. elegans* fat storage. In the worm, LDs are enriched in TAG, whereas cholesterol is mainly deposited in LROs [101]. Connecting LDs with LROs, it is speculated that LROs mediate the flux of fatty acids from LDs to either mitochondria or peroxisomes [102]. Until recently, the discrimination between LROs and LDs in the lipid management in *C. elegans* was widely neglected, and some phenotypes attributed to LDs more likely may be associated with LROs. Today, however, several specific approaches and staining protocols are available, which allow a clear distinction between these two fat-storing cell organelles. Among these, three methods should be mentioned here briefly: (1) In transmission electron microscopy, LDs appear to be electron lucent, whereas the more dense LROs appear to be electron-dense and opaque [103]. (2) Both Nile Red and BODIPY are established as vital dyes for monitoring LDs in a broad variety of organisms. In *C. elegans*, both dyes show a high affinity for LROs, whereas Nile Red fails to stain LDs in living nematodes [100]. (3) Some bona fide LD-resident proteins have been identified in *C. elegans*. One of these proteins is the triacylglycerol lipase ATGL1 which, upon fusion with GFP, specifically stains LDs but not LROs [103].

### 3.3. Lipid Droplets, Insulin Signaling, and Autophagy

As already addressed in the preceding chapter, caloric restriction represents one of the best-known and most reproducible interventions to prolong eukaryotic lifespan. This phenomenon was first observed in rodents [104], and among others was confirmed in yeast cells [81], *C. elegans* [92], *Drosophila melanogaster* [105], and primates [106]. In *C. elegans*, the intestine as a central organ of the worm is tightly linked to the aging process [107]. Therefore, nutrition is of eminent importance to *C. elegans* and all life-prolonging processes relate directly or indirectly to caloric restriction. In *C. elegans*, nutritional supply is covered by the ingestion of bacteria, and reducing the number of bacteria that are experimentally fed allows extension of the lifespan of up to 70%. This effect was observed in all phases of the worm’s life cycle (either growth, reproduction, or post-reproduction phase) [108,109]. Caloric restriction leads to obvious changes in the *C. elegans* phenotype, foremost the reduction in body size, and leads to characteristic changes in lipid metabolism as reflected by an increased TAG: protein ratio observed in L4 larvae as well as in the adult worm. As consequence, this increase in TAG levels also manifests in LD size and abundance. For the wild type, depending on body region and developmental stage, an up to 15-fold increase in the number of enlarged LDs upon caloric restriction has been reported. The very same enlargement in LDs was seen in *eat-2* mutant worms (suffering from a feeding defect) that serve as a genetic model for caloric restriction [108]. Moreover, in L2 larva, starvation can induce development to the dauer larva state. This transition is marked by fat accumulation serving as an internal energy reserve, which occurs in conjunction with a substantial increase in LD number and density in the dauer larvae [110]. The exact mechanics underlying the outcome of caloric restriction are not completely clear, but have to be considered multifactorial. Involved processes may, inter alia, comprise (i) the down-regulation of insulin/insulin-like growth factor 1 (IGF-1) signaling (IIS), (ii) a decline in TOR signaling yielding elevated autophagy, (iii) increased activation of sirtuins resulting in gene silencing, and (iv) a more complex regulated decline of the metabolic rate [111,112,113]. 

IIS-pathway/FOXO. The evolutionary highly conserved IIS pathway plays an important role in nutrient sensing and maintenance of glucose homeostasis. Central to this is the IIS-regulated expression of a set of genes involved in stress response, energy generation, drug metabolism, and chaperone activity [114]. Concerning the heavily discussed life-prolonging effect of caloric restriction, certain arguments account for a contribution of IIS, while others are conflicting, such as the additive effect of caloric restriction and IIS repression on life extension [111,113]. In fact, entry of *C. elegans* L2 larva into the dauer larva stage is blocked by activated IIS. Screens searching for mutations that promote the L2/dauer larva transition led to the identification of several IIS-pathway elements. In toto, the associated genes were given the name *daf*, as an abbreviation for “dauer formation” variant [99]. The first, upstream component of the IIS pathway is a receptor tyrosine kinase (DAF-2). Upon binding of insulin-like molecules, DAF-2 activates the PI3P pathway that comprises sequential signaling via phosphoinositide-3-kinase (AGE-1), the 3-phosphoinositide-dependent kinase 1 (PDK-1), and the serine-threonine kinases AKT-1/2. The final targets of this kinase cascade are the transcription factors DAF-16 (a FOXO transcription factor; FOXO, forkhead box O) and SKN-1 (a Nrf1,2,3 transcription factor) which, upon phosphorylation, are blocked from entering the nucleus [99,115]. Hence, reduced IIS upon caloric restriction will allow DAF16 (FOXO) shuttling into the nucleus and promotion of its activity as a transcriptional regulator. With DAF-16, a central pleiotropic mediator of cellular stress responses was identified in *C. elegans* that increases resistance against stressors such as heat or pro-oxidant regimens, but also promotes fat storage [116]. Strikingly, most of the mutations that affect genetic control of IIS in a way that terminally promotes shuttling of non-phosphorylated DAF-16 into the nucleus prolong the lifespan of *C. elegans* in a drastic way; *daf-2* [117], *age-1* [88], and *pdk-1* [118] are such examples. On the contrary, mutations in *daf-16* itself suppress the increased longevity [119]. Furthermore, either mutation or knockdown of some of these IIS-associated genes resulted in a clear increase in the cellular LD content [20,120]. This suggests an association of IIS-controlled LD biogenesis with longevity in *C. elegans*. Supportive of this, Suriyalaksh et al. showed that long-lived worms reveal a strong tendency for an increased LD content [20]. However, it is also reported that an extreme excess of LDs upon passing a certain cut-off is negatively associated with lifespan [20]. These findings perfectly match with our observations in yeast. These demonstrate that a moderate increase in LDs (achieved by overexpression of Dga1p and Lro1p) results in the prolongation of both replicative and chronological lifespan, while overloading yeast cells with oleate (i.e. monounsaturated fatty acids) resulted in super-sized LDs and a clear trend to lifespan shortening (unpublished data and [19]).

TOR pathway and nutrient sensing. Another important rheostat of caloric restriction responses is the nutrient-sensing TOR (target of rapamycin) complex that exists in all eukaryotes, from yeast to humans. As the name indicates, the central component of the TOR pathway is the serine/threonine protein kinase TOR, and it is best described in mammals (termed mTORC, mammalian TOR complex). This kinase is either associated with the binding protein *raptor* (Regulatory Associated protein of mTOR), forming the TOR Complex 1 (TORC1) or *rictor* (Rapamycin-Insensitive Companion of mTOR) forming the TOR Complex 2 (TORC2) [121]. The regulation of TOR activity is highly complex, combining several input signals, such as availability of nutrients (e.g., glucose), growth factors, amino acids, and oxygen. Of importance, active TOR/mTORC inhibits autophagy, the depletion of nutrients such as those seen under starvation conditions, but also stress-derived signals, as well as the pharmacological inducer rapamycin leading to TORC decomposition, in which the deactivation of TORC results in the de-repression of autophagy [122]. Relevant to the role of IIS in caloric restriction, mTOR signaling shares a certain cross-talk with the IIS-pathway. Underlying this is the IIS-related activation of AKT-1/2, which leads to phosphorylation and inactivation of the tuberous sclerosis complex (TSC) consisting of TSC1 and TSC2. As part of an active TSC, TSC2 serves as a GTPase-activating protein (GAP) for Rheb, a small GTPase acting as a positive regulator of mTORC1. Upon IIS/AKT-dependent phosphorylation, TSC2 becomes destabilized, rendering the TSC inactive, which results in mTORC1 activation [123]. In the same way, TORC1 activity is also regulated by TORC2, which also leads to AKT-1/2 phosphorylation [124]. In addition, TORC1 is associated with further GTPases such as the Rag GTPases RAGA and RAGC, which are controlled by glucose- as well as amino-acid-pool-dependent signaling [125]. Among the manifold downstream targets of TOR, 4E-BP (eIF4E-binding protein) and S6K1 (S6 kinase 1) are the best known. 4E-BP is an inhibitor of the eIF4E translation initiation factor 4E and, by forming a complex with eIF4E, blocks translation. Upon TOR-mediated phosphorylation, 4E-BP is released from the eIF4E/4E-BP complex and translation is initiated [126]. S6K1 is another target of TOR belonging to the AGC family of protein kinases. TOR together with PDK1 phosphorylates S6K1 and, in a progression of this kinase cascade, leads to phosphorylation of the ribosomal protein S6, resulting in the translation of specific mRNAs [127]. Most (but not all) components of these highly conserved pathways are existent in *C. elegans* and higher eukaryotes (the *C. elegans* homologues are given in brackets): TOR (LET-363); Raptor (DAF-15); Rictor (RICT-1); RHEB (RHEB-1); RAGA (RAGA-1); RAGC (RAGC-1); 4E-BP (IFET-1); S6K1 (RSKS-1) [121]. In *C. elegans*, it was shown that deletion of the TOR homolog LET-363, and as a result deletion, of the central element of the TOR pathway, leads to an arrest of the larva in the L3 stage [128]. On the other hand, the RNAi-mediated knockdown of let-363 resulted in a dramatically increased mean lifespan and elevated lipid accumulation that is seen most obviously in intestinal cells [128]. Moreover, as revealed either by gene mutation or knockdown (RNAi) experiments, the reduced expression of TOR-pathway-associated compounds RICT-1 [129], DAF-15 [90], RHEB-1 [130], RSKS-1 [131], RAGA-1 [129], and RAGC-1 [129] extends the lifespan of *C. elegans*, thus mimicking the effect of nutrient depletion/caloric restriction (for a detailed review see [121]). In addition, mutations in rct-1 [132,133] and rsks-1 [134], and a RNAi-mediated knockdown of DAF-15 [90], resulted in the formation of numerous enlarged LDs, especially in intestinal cells, with the phenotype seen in daf-15 RNAi experiments marked by the increased presence of autofluorescent granules, most probably representing LROs [90]. In this context, it appears noteworthy that the aging-dependent sequestration of protein aggregates by LDs has been reported to occur in mouse intestinal tissue, where this process may serve the removal of protein aggregates for subsequent autophagic digest via lipophagy [135]. Hence, it cannot be excluded that the appearance of enlarged LDs/LROs in intestinal cells of *C. elegans* is also connected with a similar LD/autophagy-associated process of protein aggregate clearance. The intrinsic linkage between TOR inhibition and LD synthesis seen in *C. elegans* represents an evolutionary recurring motif, as discussed below for *Drosophila melanogaster* and *Homo sapiens*, and also holds true for unicellular eukaryotes (Figure 2 and Section 2). In *S. cerevisiae*, treatment with substances such as rapamycin or methionine sulfoximine inhibits TOR signaling and promotes chronological lifespan of the yeast cells [82]. Another consequence of rapamycin exposure is an increased TAG synthesis that is accompanied by increased LD numbers [84]. However, both investigations did not show whether the observed life-prolonging effect of rapamycin in yeast cells is due to an interplay between TOR inhibition and LD biogenesis, or whether these outcomes represent independent effects of rapamycin.

### 3.4. Lipid Droplets and TGF-β Signaling

As a further pathway contributing to lifespan expansion upon caloric restriction, we discuss the influence of TGF-β signaling on longevity in *C. elegans* [136]. Five members of the TGF-β superfamily have been identified in *C. elegans*, and with respect to its implication in aging, dauer larva formation, and fat storage, we focus on the TGF-β homolog DAF-7 [137]. Produced under favorable conditions by sensory, amphid ASI neurons, DAF-7/TGF-β stimulates TGF-β receptor (TGF-βR)/Smad-based signaling. The final downstream target affected upon DAF-7 ligation in the TGF-β/Smad pathway is the nuclear factor co-SMAD DAF-3, which binds to the Sno/Ski transcriptional co-factor DAF-5 and promotes by this expression the genes responsible for dauer larva formation. Homodimeric DAF-7 binds to a heterotetramer consisting of two molecules, DAF-1 and DAF-4, resembling the TGF-βR homologous receptor localizing to the plasma membrane in *C. elegans*. In a canonical mode, DAF-7 ligation leads to activation of DAF-4, a Type II TGF-βR which phosphorylates and activates the type I TGF-βR DAF-1 that itself is a serine/threonine kinase. Downstream to this, activated DAF-1 phosphorylates the R-Smad homologs DAF-8 (Smad2) and DAF-14 (Smad8) which, upon heterodimerization, translocate to the nucleus. In the nucleus, heterodimeric DAF-8/DAF-14 inhibits the Co-factor/Co-Smad DAF-3 (Smad4) and the Sno/Ski homologous transcription factor DAF-5, and, due to this, blocks the transcription of dauer-specific genes [138,139,140]. Each intervention that blocks TGF-β/Smad signaling (i.e., mutations in daf-7, daf-4, daf-8, daf-1, and daf-14) prolongs *C. elegans* lifespan, whereas each opposite intervention boosting TGF-β signaling (mutations in daf-3 and daf-5) shortens the lifespan [141]. In animals with mutated daf-7, daf-1, and daf-4, the improved lifespan was paralleled by a 2.5-fold increased fat accumulation, most probably in LDs compared to wild-type worms. It is noteworthy that this increased fat storage was independent of a reduced food intake, but was a specific outcome of defective (inhibited) TGF-β/Smad signaling [142].

### 3.5. Significance of Lipid Droplet Accumulation to C. elegans Lifespan 

These findings together demonstrate the strong interference of aging-associated pathways with nutrient sensing (IIS, TOR signaling) and developmental growth regulation (TGF-β signaling) in controlling/blocking dauer larva transition [143]. Accordingly, inactivation of each of these pathways will support longevity by promoting the exit from normal development to the dauer larva state. Of high relevance to this, all of the “anti-aging interventions” in *C. elegans* addressed in this review were accompanied by a strong accumulation of LDs. Since the formation of dauer larvae is also inextricably linked to a shifted LD abundance, it may be questioned whether this reflects an evolutionary developed mechanism of intrinsic energy supply under poor environmental conditions and/or the degree to which LD accumulation is an active, driving force in the aging process. The following section aims at addressing this question by discussing two findings, which suggest an active role of LDs in coping with cellular stress conditions. 

The Lapierre group showed that overexpression of the autophagy receptor sequestosome (SQST-1) resulted in a decreased lifespan of *C. elegans* at 25 °C [144]. Upon applying a genome-wide RNAi screen, they identified candidates that were able to alter the protein content of a SQST-1-GFP fusion protein, and the candidate list revealed a huge overlap with proteins that were found to be part of the LD proteome of nematodes. In order to boost the cellular LD content, the atgl-1 lipase was silenced, which shifted LDs in numbers and size, and, as a result, replicated observations frequently made in long-lived worms. This increase in lipid storage was accompanied by a strong lifespan extension and accumulation of SQST-1 at LDs. Interestingly, the SQST-1 relocalization to LDs was not restricted to this autophagic receptor, but was also observed for misfolded as well as ubiquitinylated proteins, suggesting a general role of LDs in protein homeostasis [144]. This matches perfectly with our observations made in yeast cells, showing that stimulating LDs can prolong both the replicative and chronological lifespan, most likely by detoxifying harmful proteins [19,35].

Independent of caloric restriction, but the same as in *S. cerevisiae*, there appears to be an interplay between lipid droplets and the ER-associated degradation machinery (ERAD). It was shown that an oleate-rich diet stimulates LD levels, ERAD activity, and longevity in *C. elegans.* The life-prolonging effect of oleate was also strongly dependent on LD-associated proteins such as Plin-1 and Fitm-2 (fat-storage-inducing transmembrane 2) [145]. Plin-1 is the only known perilipin in *C. elegans* [146,147], whereas Fitm-2 is essential for the budding of LDs from the ER [148]. Another publication also confirmed the life-prolonging effect of monounsaturated fatty acids such as oleate [149].

As seen in most eukaryotes, a fraction of LDs can reside in the nucleoplasm; in *C. elegans*, such nuclear LDs are found in the nuclei of intestinal cells, especially under stress conditions [89]. Some of these LDs were shown to be covered by heterochromatin, which is translocated apart from the nuclear lamina. Mosquera et al. speculate that this inward movement of heterochromatin could result in relieved gene silencing and, as a result, promote the aging process. Furthermore, the authors also reported that giant nuclear LDs may come in close contact with the nuclear lamina, especially in areas devoid of lamina. According to the authors’ assumptions, these giant LD/nuclear lamina-contact zones could be responsible for ruptures of the nuclear lamina, which are seen frequently in *C. elegans* intestinal cells [150]. Although this may hold true, it is tempting to speculate in a different direction by considering the lamina/nuclear LD/chromatin association areas as sites specialized for chromatin/DNA repair.

In the model organism *Drosophila melanogaster*, with the development of specialized cells and tissues, the evaluation of the role of LDs in the aging process is much more difficult, but here, too, a clear interconnection between lifespan extension, LDs, mTOR, and IIS is beginning to emerge, as the following section shows.

## 4. Lipid Droplets in *Drosophila melanogaster*

The fruit fly *Drosophila melanogaster* is a well-described animal model organism in genetics, developmental biology, and cell and molecular biological research on the mechanics of senescence and aging [151,152]. Throughout evolution, the “hallmarks” defined for mammalian aging [74] are highly conserved and can also be investigated in *Drosophila*. Consequently, studies in flies identified evolutionary conserved gene mutations, endocrine and cellular signaling mechanisms, and tissue- and environment-specific factors including their interactions with the genetic background, that affect lifespan [153]. Focusing on the metabolic aspect, several lipid-metabolism-associated contexts of substantial physiological and pathophysiological relevance have been addressed in *Drosophila*. These comprise research on TAG storage and mobilization from LDs that have been addressed in *Drosophila*, which also reflect lipid metabolism in humans, including age- and lipid-associated diseases. As will be outlined in this section, evidence is increasing that LDs are deeply involved in the interconnection of nutritional, metabolic, and stress-associated signaling, emphasizing their role as cell organelles with multifaceted implications in lifespan control. 

### 4.1. Lipid Droplets and Drosophila Development 

Development of the fruit fly proceeds in an indirect mode, with each developmental stage (egg/embryo–larva–pupa/metamorphosis–imago) differing under nutritional aspects. Lipid homeostasis is regulated in a food-dependent mode during the larval-hood and in the adult fly (imago). In contrast, “nutritional supply” for embryogenesis depends on the maternal deposition of LDs during oocyte maturation, and the energy needed for metamorphosis (pupa, imaginal disc development) is supplied by the LD-rich fat body established during the larval stage, which shares functional equivalence with the mammalian liver and adipose tissue. Reflecting the adverse effects of diet-associated obesity in higher organisms predisposing for a number of pathological conditions, excessive consumption of a high-fat diet decreases lifespan in *Drosophila* [154]. In line with this, *Drosophila* mutants devoid of adipokinetic hormone *Akh* (a functional analog of glucagon), serving as a genetic model of obesity, suffer from lifespan shortening accompanied by characteristic, age-dependent changes in the lipid profile that especially affects the TAG signature [155]. This accounts for a selective TAG degradation from LDs in moribund flies yielding a senescence-specific lipid signature.

As stated above, LDs play crucial roles during all stages of *Drosophila* ontogenesis. For instance, oocytes are loaded with TAG-rich LDs to comply with the metabolic demands of embryogenesis [156,157,158,159]. This maternally driven process is regulated by the *Drosophila* perilipin 2 (PLIN2) homologue LSD-2 (lipid storage droplet 2) [160,161,162]. Beyond this, LSD-2 and LSD-1, the homologue of human perilipin 1(PLIN1) [163], act as central regulators of LD growth and fat storage over the whole lifespan of *Drosophila* [164]. During the larval stage, LDs are indispensable to fat body growth, which is controlled via metabolic signaling involving the IIS/FOXO (dFOXO in *Drosophila*) pathway as well as endocrine signaling [165,166,167]. Moreover, LDs connect fat body growth with molting, since synthesis of ecdysone (a precursor of the molting regulatory steroid hormone 20-hydroxyecdysoen) requires cholesterol trafficking from LDs to autophagosomes, cholesterol-rich LDs accumulating in the larva if autophagy is inhibited by the accumulation of fat [168]. Larval development is influenced in a nutrition-dependent mode by the LD-associated protein CG9186/Sturkopf, which regulates larval growth by connecting LD biogenesis to nutritional supply via interaction with the IIS/dFOXO pathway and hormone (juvenile hormone) signaling [169]. In the absence of LDs, the CG9186/Sturkopf protein localizes to the ER, but translocates to LDs upon induction of lipid storage [170]. Interestingly, in *Drosophila* CG9186/Sturkopf null mutants, TAG storage is neither affected in embryos (laid down by mutant mothers) nor in mutant larva containing normal LDs, an effect that is not completely understood at present, but could be based on distinct dFOXO targets such as the *Drosophila* lipase *brummer*, an antagonist of LD-regulatory LSD-2 (see the next section) [169]. In contrast, CG9186/Sturkopf null mutations become manifest only in adult flies, which show a markedly reduced TAG storage. In addition, adult CG9186/Sturkopf mutants also reveal a markedly increased protection from desiccation stress (supposedly due to a changed hydrocarbon composition of the cuticula) and reduced locomotor activity [169]. Moreover, as we discuss below, the reduced LD content seen in adult CG9186/Sturkopf mutants negatively affects stress resistance as well as lifespan of the adult flies. 

### 4.2. Control of the Lipid Droplet Pool in Drosophila Adipocytes 

Interestingly, *Drosophila* adipocytes bear spatially and functionally distinct LD pools that access distinct lipid pools for their individual maintenance [171]. Larger LDs, residing in the central cell body, require supply by fatty acid synthetase FASN-1 de novo lipogenesis, whereas smaller LDs locating to the cell periphery require gut-derived lipophorin shuttle (Lpp)-dependent lipid supply. The population of small peripheral LDs stays in direct contact with the plasma membrane and its organization changes during fasting periods. This starvation-associated effect is regulated by the protein *Snazarus*, which binds LDs at the ER-plasma membrane contact sites via a C-Nexin domain. TAG storage is enhanced upon Snazarus overexpression, which confers resistance to starvation conditions and yields lifespan prolongation [171]. Conversely, starvation also leads to the up-regulation of the *Drosophila* lipase *brummer* (bmm), an orthologue of mammalian adipose triglyceride lipase, which binds to LDs via a so-called brummer domain and elevates TAG mobilization from LDs [157]. Under normal feeding conditions, loss of bmm activity causes a moderate lifespan reduction, with the mutant flies developing an obese phenotype with adipocytes containing markedly enlarged LDs. In contrast, starving bmm mutants show a marked lifespan extension [157], which is considered to be due to the decelerated TAG mobilization from LDs [172], albeit the involvement of other lipases such as *doppelgänger von brummer* (dob) or other putative starvation-induced lipases may also play a role [157]. 

As mentioned before, the LD pool of *Drosophila* adipocytes is controlled by LSD-1 (PLIN-1) and LSD-2 (PLIN-2), which affect bmm lipase activity in opposite directions. While LSD-1 supports lipolysis by recruiting bmm to LDs [173], LSD-2 antagonizes bmm access and protects LDs from TGA mobilization [173]. Conversely, binding of bmm to LDs under starvation conditions antagonizes the anti-lipolytic activity of LSD-2 [157]. Concerning the LD phenotype, LSD-2 mutants show no peculiar alterations [164]. In contrast, LSD-1 deficiency promotes LD accumulation [168], with the adipocytes developing a reduced number of markedly enlarged, “giant” LDs during the larval hood and in the adult fly [163]. Moreover, it is clearly shown that the lifespan of LSD-1 mutants is reduced under starvation conditions [173]. Bi and co-workers proposed that LSD-1 and LSD-2 regulate lipolysis in an opponent, LD-size-dependent mode, with LSD-1 promoting TAG mobilization from large LDs, but LSD-2 protecting small LDs from bmm-induced lipolysis [173]. The same study also demonstrated that LSD-1 is able to adopt the anti-lipolytic property of LSD-2 under certain conditions, which points to a functional redundancy between these perilipin homologs in *Drosophila*. 

LSD-2 affects the *Drosophila* LD pool already at the earliest developmental stages, with the oocytes of LSD-2 mutant females showing a substantially reduced TAG content, causing impaired embryogenesis [162]. Similarly, the fat body of LSD-2^−/−^ homozygous larva contains less than 75% TAG as seen in normal flies. Pointing to additional functions not directly related to lipid storage, LSD-2 is also required for the endoreplication of cells in the larval salivary gland, with the loss of LSD-2 activity resulting in enhanced ROS generation and stimulation of JNK (c-jun amino-terminal kinase)-dependent apoptotic cell death [174]. Similarly, LSD-2 provides lipid storage in imaginal discs during metamorphosis, but also plays a distinct role in imaginal disc cell growth and differentiation. For instance, LSD-2 expression is stimulated in the wing imaginal disc upon overexpression of the gene vestigial (vg), which encodes a transcription factor controlling wing cell proliferation and differentiation [175]. Correspondingly, knockdown of LSD-2 promotes cell death in the wing imaginal disc, which involves the dFOXO-dependent up-regulation of pro-apoptotic *reaper* [176]. Notably, *reaper* is an orthologue of human autophagy/mTOR-regulatory TSC1 (see Section 3.3) and stimulates apoptosis in *Drosophila* by suppressing anti-apoptotic Diap1 [177]. Diap1, in turn, represents the orthologue of mammalian caspase-activation-inhibiting XIAP, which connects developmental (TGF-β1/BMP) and (oxidative) stress-associated (NF-κB and Nrf2) signaling with apoptosis in mammalian cells [178,179,180]. These findings point to a distinct role of LDs and LD-associated perilipins (LSD-1, LSD-2) in cellular growth control, interconnecting metabolic regulation with cell cycle/cell death checkpoints, which also affects *Drosophila* lifespan. As already stated, this was shown for LSD-1 under starvation conditions [173] and the involvement of LSD-2 in lifespan control under a high-fat diet has also been discussed [181]. 

### 4.3. Lipid Droplets and Lifespan Extension in Drosophila 

Complementary to studies on *Drosophila* lipid/LD biology, further investigations addressed the effects of caloric restriction, pharmacological intervention, and the combination of both on the genotype–environment interaction and the underlying mechanisms in *Drosophila* [182,183,184]. In agreement with the above-mentioned lifespan-shortening effect seen for the Akh-deficiency-based genetic model of obesity [155], these studies revealed a positive effect of dietary restriction and nutritional balance on longevity in *Drosophila*, for which nutrient sensing and the associated downstream signaling is of pivotal relevance [184,185]. Similar to the role of IIS/FOXO in *C. elegans* dauer larva transition and, as stated, for the involvement of CG9186/Sturkopf in *Drosophila* development, the IIS-dependent regulation of dFOXO is also involved in *Drosophila* lifespan control. This is indicated by the finding that reduced IIS (as seen upon caloric/dietary restriction) results in enhanced dFOXO activity and lifespan extension in the fly [186,187]. The IIS-dependent effects are largely mediated by the JNK/dFOXO stress response pathway [187,188,189,190] and other transcription factors acting downstream of dFOXO such as AOP (Anterior open, an E-twenty-six (ETS)-family transcriptional repressor), which, in a coordinated manner, mediate the lifespan extension in *Drosophila* [191]. It is noteworthy that dietary inputs address organ-specific interactions. For instance, overexpression of dFOXO in the fat body of the head leads to an extended lifespan only under high protein conditions [192]. Interestingly, flies bearing lifespan-extending deteriorations in IIS, such as those caused by the deletion of insulin-like peptide 2, show an increased body fat storage and are more resistant to lipophilic toxins and oxidative stress [193]. In sum, the regulatory network mediated by IIS that controls LD size and number is highly complex. In *Drosophila* nurse cells (which dump LDs to the oocyte), strong IIS stimulation, e.g., due to the loss of the IIS antagonist PTEN (phosphatase and tensin homologue), leads to the accumulation of abnormally enlarged LDs [194,195]. This is counterbalanced by dFOXO, which induces lipases such as *brummer* [196], which is antagonized by LSD-2, protecting LDs from bmm lipase access as explained above. In this context, it is worthwhile to mention again that LSD-2 can only be found on small LDs, whereas LSD-1 can anchor to LDs of different sizes [173]. Hence, IIS/dFOXO-dependent responses are specifically controlled at the LD level by LSD-1 and LSD-2. 

Moreover, excessive intestinal stem cell (ISC) proliferation causing intestinal dysplasia in aged flies is suppressed by reduced IIS/JNK signaling, which restores proliferative homeostasis and extends *Drosophila* lifespan [197]. Connected with this, ER stress-associated UPR (UPR^ER^, see Section 2) has been shown to also play an important regulatory role in ISC proliferation, with the chronic UPR^ER^-dependent hyper-stimulation of ISC proliferation being causal to age-related intestinal dysplasia [198]. Central to this UPR^ER^ response in ISC proliferation in *Drosophila* is an orchestrated interplay involving (i) the activity of PKR-like ER kinase (PERK) which is controlled by the JAK/Stat pathway, (ii) transcriptional control via Ire1 (endoribonuclease 1)-mediated splicing of the transcription factor Xbp1 (X-Box binding protein 1), and (iii) the activation of another transcriptional regulator ATF6 [198,199]. With respect to lipid metabolism, UPR^ER^-dependent Ire1/Xbp1 signaling is of special relevance since it connects ER stress to triacylglycerol synthesis and lifespan extension in *Drosophila* [200]. Upon caloric restriction, Ire1/Xbp1 signaling promotes lipogenesis and TAG accumulation in intestinal enterocytes and prolongs lifespan of the fly; this effect also involves activity of the transcription factor *sugarbabe* (a Gli-like zinc finger transcription factor involved in the carbohydrate metabolism). With respect to this, the concept that an Ire1/DGAT2-based shift of LD biogenesis, such as that seen under conditions of ER stress in the mouse liver, could improve LD-mediated protection from phospholipid oxidation [47] (see Section 5), is of particular interest. Indeed, LDs have been shown to confer antioxidant properties in *Drosophila* by incorporating TAG redistributed from PUFAs and, as a result, protect PUFAs from lipid peroxidation in neural glial cells [201]. Hence, it would be interesting to investigate whether aging- and/or starvation-induced ER stress leading to the stimulation of Ire-1 and Xbp1-dependent LD biogenesis also confers lifespan extension in *Drosophila* by improving the anti-oxidative capacity. If so, however, this protection will demand tight control of LD abundance since the ROS-stimulated accumulation of LDs is at risk of promoting neurodegeneration in *Drosophila* [46]. According to this study, ROS generation emerging from mitochondrial dysfunction is causal to glial LD accumulation, a finding that is considered to indicate an evolutionary conserved process of pathogenic relevance to neurodegeneration. It is tempting to speculate whether harmful mitochondrial proteins can also be shuttled to LDs in *Drosophila*, as shown in yeast [7]. Pointing in this direction, analysis of the LD proteome in embryonic [202] and adult [203] tissue in *Drosophila* revealed the presence of mitochondrial proteins in LDs. In addition, similar to the binding of protein-aggregate-enriched IBs by LDs in yeast [8], protein aggregates formed upon ER stress/oxidative stress could also be sequestered by specific LD binding and, as a result, confer protection in the neuroglia. In fact, LD binding of protein aggregates has been demonstrated recently for mouse intestinal tissue which may be followed by lipophagic digest [135]. Thus, a similar process could contribute to the degradation of protein aggregates via lipophagy in neuroglia, but also other tissues, such as the intestine in *Drosophila.*

Autophagy–TOR pathway. Similar to *C. elegans*, the autophagy–regulatory TOR pathway is also intimately involved in metabolic homeostasis and lifespan control in *Drosophila* [204]. In general, the inhibition of mTOR signaling results in lowered translational activity and elevated autophagy which improves proteostasis [205], a critical hallmark of aging [74]. It should already be mentioned at this point that, in *Drosophila*, both nutrient deficiency and TOR inhibition also lead to an increase in LD size [206]. The necessity of enhanced, functional autophagy for lifespan extension in *Drosophila* was demonstrated by feeding experiments in adult flies using the TOR inhibitor rapamycin, and it was shown that the stimulation of autophagy resulted in prolonged survival of starving wild-type animals but also enhanced the lifespan of *Drosophila* IIS mutants [207]. Moreover, this investigation also demonstrated that rapamycin-induced autophagy also improves the resistance towards paraquat (1,1′-Dimethyl-4,4′-bipyridin)-induced oxidative stress. Paraquat serves as an insecticide via the cytochrome P450/Fenton-reaction-dependent formation of free hydroxyl radicals [208], which impair intestinal regeneration and cause substantial cell damage in the aging fly [170]. Revealing the involvement of LDs, flies with a reduced LD content such as that found in adult CG9186/Sturkopf mutants show reduced survival and a decreased lifespan upon paraquat treatment [169]. Pointing further to the role of autophagy, *Drosophila* mutants lacking a proper autophagic flux due to mutation of the autophagy–regulatory gene Atg8a (autophagy-related 8a) show a shortened lifespan accompanied by enhanced protein oxidation and ubiquitination, effects which are aggravated by pro-oxidant conditions [209]. Contrarily, the age-dependent decline in autophagy seen in *Drosophila*, especially in the nervous tissue as a result of reduced Atg-expression, is counteracted by Atg8 overexpression, which improves oxidative stress tolerance and longevity in aged flies. As stated above, the transfer of cholesterol from LDs to Atg8-rich vesicles (autophagosomes) is essential to larval hormone synthesis, with the up-regulation of TOR limiting Atg8 expression and autophagosome formation, which leads to LD accumulation [168]. With respect to this, it is tempting to hypothesize about a similar Atg8-related mechanism shifting LD numbers in the adult fly in support of cell surveillance if autophagy declines. In addition to Atg8, a connection with increased longevity and enhanced stress responses was reported also for other compounds, enhancing autophagy especially in the nervous system of *Drosophila*. These include AUTEN-67 and 99 (autophagy enhancer-67 and -99), acting downstream of TOR at the level of autophagosome membrane formation [210,211] and spermidine, which elevates autophagy upon interference with epigenetic control and yields lifespan expansion in yeast, *C. elegans*, *Drosophila*, and human cell lines [210,211,212].

Furthermore, autophagy is also stimulated by nutritional factors such as flavonoids, a class of plant polyphenolic compound with well-known antioxidant properties [213]. Among these, isoquercetin and xanthohumol have been proven to boost LD formation in *Drosophila*, especially in cells of the nervous system [214]. In addition, xanthohumol was shown to increase the resistance of adult flies to several stressors (e.g., hydrogen peroxide, paraquat, starvation, and heat) and prolong lifespan in *Drosophila* [215]. As discussed below, flavonoids also exert similar protective, antioxidant effects in the human system [216]; for instance, quercetin counteracts liver steatosis by inhibiting lipid peroxidation [217]. 

As mentioned above, TOR (mTOR) signaling directly affects the translational activator S6 kinase [204,207] and the translational repressor 4EBP [207,218,219]. In *Drosophila*, additional nutritional sensors have been described that modulate lifespan, including the transcription factor ATF4 [220], amino acid deprivation–activated kinase GCN2 acting upstream of ATF4 [221], GCN2 deficiency leading to a massive loss of TAGs and thus LDs [222], and AMP-activated protein kinase (AMPK). From the mechanistic point of view, AMPK acts as a central, positive regulator of autophagy by inhibiting mTORC1 (via phosphorylation of TSC-associated TSC2). AMPK extends the lifespan of adult flies in an autophagy-dependent mode, specifically affecting the central nervous system and intestinal tissue [223,224]. In contrast, reduced AMPK activity is associated with an enhanced susceptibility to starvation-induced lethality (especially in the larva; AMPK null mutants are larval lethal) and abnormal lipid accumulation marked by the accumulation of enlarged LDs in larval tissue [225]. Of note, this LD-related phenotype is seen in normally fed, mutant larva lacking AMPK activity and resembles the phenotype observed in oenocytes of starving wild-type larva [226]. Oenocytes are the primary site of LD accumulation seen in starving *Drosophila* larva and, analogous to the role of human hepatocytes in systemic lipid homeostasis, are sites of lipid release from the larval fat body during starvation. Strengthening the crucial role of autophagy on lifespan control, rapamycin partially improved the survival of adult flies with reduced AMPK activity under starvation conditions. Interestingly, the type-2 diabetes therapeutic compound metformin, which antagonizes IIS but stimulates AMPK signaling, and, as a result, also shifts autophagy (recently reviewed in [227]), extended lifespan in *C. elegans* and healthy mice [228,229]. However, Slack et al. reported that metformin failed to prolong lifespan in adult *Drosophila*; at the same time, the metformin-mediated activation of AMPK resulted in a drop in TAG levels [230]. With respect to this, it is noteworthy that metformin inhibits TOR in *Drosophila* independently of AMPK stimulation [231] and Slack et al. suspected a potential negative interference of metformin effects, not related to AMPK, with positive effects of AMPK activation on *Drosophila* lifespan [230]. Support to this is provided by the finding that overexpression of the serine/threonine kinase LKB1, another positive regulator of AMPK, indeed extends lifespan in Drosophila [232]. LKB1-null mutant flies with reduced AMPK activation show decreased TAG levels, a phenotype that can be compensated by transgenic expression of wild-type AMPK [233]. Accordingly, this suggests that AMPK activation at reduced LD numbers does not affect lifespan, whereas AMPK activation at an increased LD abundance extends the lifespan in *Drosophila*. 

### 4.4. Lipid Droplets, Transsulfuration, and Cellular Antioxidant Defenses

Together, these findings emphasize the pivotal role of metabolic regulation and LD homeostasis in lifespan determination. At its most basic, this applies to the maintenance of metabolite pools, especially under dietary restriction [134], and in particular to the amino acid balance, which is controlled on the anabolic (translation) and catabolic (protein degradation, autophagy) levels as a fundament of cellular proteostasis, with its dysregulation representing another hallmark of aging [74]. This was demonstrated in a study by Grandsion et al., which shows that dietary-restriction-based lifespan extension in *Drosophila* is abolished by feeding the flies a mix containing all essential amino acids (EAA feed), while using an EEA-feed omitting methionine does not affect starvation-induced lifespan prolongation [234]. Further research revealed that trans-sulfuration (i.e., the production of cysteine from methionine-derived homocysteine or cystathionine) plays an important role in extending survival under starvation conditions in *Drosophila* [235]. Upon dietary restriction, enhanced trans-sulfuration preserves lipid storage in LDs (to levels seen in fully fed flies) and, due to the excessive consumption of methionine, lowers overall protein synthesis. With respect to this, the positive effect of limited methionine availability on lifespan seems paradoxical. However, the beneficial effect of methionine restriction on longevity is conserved from yeast to mammals as demonstrated by the improved longevity seen in yeast, *Drosophila*, and rodents upon sulfur-amino acid starvation (SAAR, also referring to cysteine) [236,237,238,239]. Of particular relevance to this, in yeast, methionine starvation contributes to longevity by stimulating autophagy and an improved vacuolar acidification [239]. This puts emphasis on the specific role of sulfur-containing methionine and cysteine pools in cellular responses to nutritional stress and aging, primarily acting on the autophagic flux. Similar to starving flies in which inhibition of trans-sulfuration (resembling SAAR) lowers TAG levels [235], dietary SAAR reduces fat deposition and the TAG content in the rodent liver [240,241]. Stressing the aspect of autophagy, SAAR acts on the same metabolic regulators in the mammalian system—GCN2, ATF4, and AMPK (for a review see [236])—that link nutritional sensing to the onset of autophagy, and act as lifespan modulators in *Drosophila* as outlined above.

Pointing to a further, indirectly nutrition-associated aspect of profound relevance to lifespan control, the extended survival seen in rodents upon SAAR is accompanied by increased systemic levels of the cysteine-containing antioxidant glutathione (GSH) [237]. This agrees well with the inverse correlation existing between aging and systemic GSH levels [242]. In addition, GSH levels are also elevated in long-lived flies upon dietary restriction [235], an effect that is attributable to the trans-sulfuration-based refueling of the cellular cysteine pool. Explaining this, dietary restriction up-regulates the expression of cystathionine β-synthase (CBS), a key enzyme catalyzing the conversion of homocysteine to the cysteine precursor cystathionine, and elevation of CBS synthesis is essential to lifespan extension in the starving fly [235]. In good accordance with this, blocking the final step of trans-sulfuration-based cysteine synthesis, which is catalyzed by cystathionine-γ-lyase, leads to a marked drop in GSH levels and abolishes the starvation-based lifespan–extension in *Drosophila* [235]. Connecting the trans-sulfuration pathway to LD biogenesis, inhibition of cystathionine-γ-lyase also lowers the overall LD content in *Drosophila* [235]. Of note, this effect of trans-sulfuration inhibition on LD abundance seems to represent an evolutionarily conserved motive, since in human ovarian cancer cells the knockdown of CBS also results in reduced LD numbers [243]. Taking into consideration the crucial role of the GSH/GSSG (the oxidized GS=SG disulfide) redox balance in cellular antioxidant capacity, the elevation of trans-sulfuration has to be considered pivotal to cellular stress adaption by connecting metabolic competence to pro-/antioxidant balance. Accordingly, it makes sense that an increase in GSH is associated with an increase in LDs under oxidative stress. Considering the pivotal role of GSH in cellular detoxification of peroxides (H_2_O_2_, but also lipoperoxides) and lipid-peroxidation-derived metabolites such as 4-hydroxy-2-nonenal (HNE), limitations of GSH availability, ensuing from enhanced GSH consumption and/or inadequate GSH synthesis, are at considerable risk of promoting oxidative damage to lipids, DNA, and proteins [244,245]. Hence, LDs could support cell survival under conditions of GHS depletion by eliminating oxidized lipids and misfolded proteins as discussed above. Serving a similar, cytoprotective task, LDs have been shown to adopt the role of a cellular antioxidant in *Drosophila* larva by sequestering polyunsaturated fatty acids from the cell membranes, which yields protection of these lipids from peroxidation [201]. 

Moreover, as a sulfhydryl group donor, methionine also undertakes the synthesis of iron–sulfur clusters (Fe-S) via the mitochondrial ISCU (iron–sulfur cluster forming unit) [246]. Fe-S clusters are indispensable for electron transfer in the respiratory chain and energy charge in all aerobic organisms. In addition, Fe-S clusters are essential to the citrate dehydrogenase activity of aconitase and are co-factors of DNA repair enzymes [247,248]. Interestingly, ablation of ISCU-mediated Fe-S biogenesis leads to increased citrate concentrations, generated from glucose-derived acetyl CoA, an elevated fatty acid synthesis, and the accumulation of LDs in human HEK293 embryonic kidney cells [249]. Furthermore, in a mouse model of Friedreich’s Ataxia (FRDA), an autosomal recessive disease marked by substantially reduced levels of the mitochondrial ISCU regulatory protein frataxin (Ftx) [250,251], the absence of Ftx function resulted in Fe-S protein deficiency, mitochondrial iron accumulation, and increased LD abundance in cardiac muscle cells [252]. Similar effects were seen in a *Drosophila* model for FRDA, where the Ftx deficiency stimulated both fatty acid synthesis and lipid peroxidation, as well as LD accumulation in glial cells [253]. Both findings suggest that hampered Fe-S cluster synthesis caused by Ftx deficiency leads to a disturbance of lipid homeostasis. However, it should not be overlooked that the FRDA/Ftx deficiency also shifts the iron content of mitochondria as demonstrated by the FRDA mouse model. It is well known that labile iron (i.e., free Fe^2+^) acts as a central cellular source for the Fenton reaction-based generation of hydroxyl radicals. In turn, these hydroxyl radicals readily react with polyunsaturated fatty acids (PUFAs) and, as a result, initiate the lipid peroxidation chain reaction (LPO), causing potentially lethal cellular damage, which is aggravated by the geno- and cytotoxic properties of LPO metabolites such as malondialdehyde and HNE [254,255,256]. Therefore, the LD accumulation seen in FRDA/Ftx-deficiency could indicate the specific up-regulation of LD biogenesis as cytoprotective response to iron-mediated oxidative stress. Support to this come from the recent finding that LDs participate in cellular responses to the pro-oxidant effects of paraquat (also a source for Fenton chemistry-based hydroxyl radical formation) in *Drosophila* [257]. It was shown that the RNA binding protein *Spen* (Split ends; the *Drosophila* orthologue of SPEN/SHARP, a regulator of NOTCH signaling) modulates the LD content in adult glial cells and provides protection from paraquat cytotoxicity. Conversely, LD biogenesis can also be stimulated by iron deficiency, which was demonstrated in human ARPE19 retinal pigment epithelium cells treated with the iron chelator deferiprone (DFP) [258]. DFP induces marked changes in lipid metabolism, including enhanced TAG synthesis, and leads to the accumulation of LDs in proximity to mitochondria followed by mitophagy. Diacylglycerol O-acyltransferase 1 has been identified as a stimulus of LD biogenesis under conditions of iron depletion, enabling the re-esterification of fatty acids regenerated upon macroautophagy [258]. 

In summary, these findings link metabolic homeostasis to stress tolerance and shed light on a particular role of LDs in cellular antioxidant defense and cytoprotection also affecting longevity. Evidence exists that the LD-mediated clearance of oxidized compounds is essential to this task, as suggested by the sequestration of LPO-products by LDs in *Drosophila* protecting larval tissue, and especially neuroblasts in imaginal discs, from hypoxia-induced oxidative stress [201]. Therefore, LDs could participate in cellular maintenance under pro-oxidant conditions by acting as a sink for lipid peroxidation products and other oxidized cellular compounds. However, considering the transient, dynamic nature of LDs, such “sinks” do not necessarily need to resemble long-term deposits for the potentially harmful “oxidized waste”. Findings in glial cells of the *Drosophila* eye point in this direction. In these cells, loss of the metalloproteinase ADAM17, a trigger of tumor necrosis factor (TNF)-based signaling, as well as lack of TNF and the *Drosophila* TNF receptor homologue *Grindelwald* [259], causes an age-related degeneration of neuronal and glial cells [260]. In this case, accumulation of LDs in glial cells prior to the degradative process confers an initial protection from glia- and neuron-derived ROS, while the subsequent metabolic decomposition of LDs leads to the release of toxic lipid peroxides causing cell damage and neurodegeneration. Hence, LDs may exert opposing effects in oxidative stress/LPO-dependent contexts, with the outcome being strongly dependent on additional factors such as nutritional sensing and lipid turnover, as well as different stress qualities (also in terms of stress duration: short, intermittent, or chronic), linking the protective capacity of LDs to the aging process.

### 4.5. Lipid Droplets and (Epi)Genetic Control

Addressing a further aspect of LD biology that may also be connected to cytoprotection, LDs may locate to the cytosol as well as to the cell nucleus. As already stated for *C. elegans*, nuclear LDs (nLDs) exist in many organisms, including *Drosophila*. Analysis of the nLD-proteome isolated from rat liver identified a number of proteins, including histones, cytoskeletal elements (e.g., cytokeratins), proteins involved in transcriptional and translational control, protein folding, and post-translational modification, and lipid-metabolism-associated carboxylesterase 1d (Ces1d; cholesteryl-ester hydrolase) [261]. Among different functions in lipid metabolism, mammalian Ces1d contributes to cellular detoxification by hydrolyzing lipid esters, either derived from xenobiotic or endogenous sources, especially in the liver and intestine [262]. With respect to LD biogenesis, Ces1d deficiency has no effect on cytosolic (ER-based) LD formation itself, but yields increased numbers of small-sized cytosolic LDs, an effect that is attributable to a lower lipid transfer to LDs [263,264]. Referring to the presence of Ces1d in nLDs in *Drosophila*, carboxylesterases may also participate in nLD biogenesis. In mammalian cells, nLDs are formed either de novo at the inner nuclear membrane or upon translocation of cytosolic LDs, originating from ER resident lipoprotein precursors, to the nucleoplasmic reticulum terminally moving to the nucleoplasm (recently reviewed by Fujimoto [265]). In hepatocytes, ER stress promotes LD shuttling to the nucleus and requires the activity of promyelocytic leukemia protein (PML) locating to the inner nuclear membrane. PML, which is critical to nuclear signaling, can be retained in nLDs (then termed lipid-associated PML structures; LAPS) and due to the PML binding properties, nLDs/LAPS may regulate the PML-mediated control of the gene- expression, for instance, as part of lipid stress responses [265,266]. The above-mentioned association between stress responses and nLDs in *C. elegans* (see Section 3) may point in a similar direction and it cannot be excluded that nLDs assist cytosolic LDs in directing the expression of lipid-metabolism-linked genes. Moreover, the association between nLDs and heterochromatin seen in *C. elegans* under stress conditions could also indicate a functional role of nLDs in aging-related gene silencing [118]. Indeed, aging in *Drosophila* is associated with changes in heterochromatin structure yielding a repression of gene silencing, which also activates the expression of transposable elements (TEs) residing in heterochromatic areas of adipocyte nuclei in the fat body (as mentioned, the *Drosophila* equivalent to the human liver) and brain tissue [267,268]. Antagonistic to this, dietary restriction counteracts TE activation and extends lifespan. Similarly, mutation of the *Drosophila* gene *Argonaute 2* (Ago2), a regulator of TE silencing [269], results in enhanced TE expression, impaired neuronal function, and reduced longevity [270]. Of note, the enhanced TE expression seen in the aged fat body of old flies is accompanied by increased DNA damage and declined levels of the nuclear-lamina protein lamin-B, with the depletion of lamin-B yielding a similar phenotype (enhanced TE expression and DNA damage) in the fat tissue of larva and young adults [267]. These findings shed light on the critical role of the nuclear lamina in gene silencing, including TF expression and genome/DNA integrity surveillance in lifespan control. 

Interestingly, in early *Drosophila* development, binding of extranuclear histones to cytosolic LDs enables the storage of histones required for chromatin organization, a task that is conferred by the protein *Jabba* [18,271]. Extranuclear histone stores are specific for very early, syncytial blastoderm stages of *Drosophila* embryogenesis that are marked by a rapid series of consecutive nuclear divisions (without accompanying cell divisions, thus forming a syncytium), with the Jabba-based recruitment of histones to LDs providing an adequate extranuclear histone supply. Although this mode of *Jabba-*aided histone-to-LD binding may be specific for early *Drosophila* embryogenesis, it is tempting to speculate that similar histone–LD interactions play a role in aging-associated changes in chromatin organization in adult flies as well as higher organisms. These may comprise chromatin remodeling and histone methylation, which are both considered further hallmarks of aging [74]. In *Drosophila*, repressive histone methylations contribute to heterochromatin stability and their disruption results in lifespan shortening [272,273]. Histones found on LDs in *Drosophila* include histones H2A and H2B, which both show an age-specific ubiquitination [18]. Reduction of the ubiquitinated form of H2A prolongs the flies’ lifespan [274]. Notably, binding of H2A by LDs would naturally serve the same purpose. In H2B, the ubiquitination is a prerequisite for the trimethylation of the histone H3K4 [18], which upon trimethylation (H3K4me3) promotes the aging process in *Drosophila* [275]. Accordingly, the possible binding of H2B by LDs would lead to a reduced amount of H3K4me3 and thus prolong the lifespan of the flies.

Moreover, the aging-associated loss of histones (originally identified as a lifespan-restricting determinant in yeast by Feser et al. [276]) and changes in the eu-/heterochromatin ratio are linked to changed histone methylation patterns that affect gene expression and may promote aging-associated DNA damage [277,278]. This fits well with the afore-mentioned *Argonaute 2* mutant phenotype in *Drosophila* marked by lamin depletion, enhanced TE expression, and DNA damage. Similarly, down-regulation of lamin-B1 accelerates the senescence of proliferating human fibroblast cells and has a profound impact on chromatin structure and gene expression [279]. Finally, it has been demonstrated that recruitment of the chromatin remodeler SNF2h (enhancing DNA accessibility in DNA repair) by *sirtuin* (SIRT6), which deacetylates histones, protects human and mouse cells from genotoxic damage [280]. On the other hand, SIRT6 also serves as a negative regulator of lipid metabolism [281], with the EGF-dependent down-regulation of SIRT6 (FOXO3/SIRT6) resulting in enhanced LD biogenesis in human colon cancer cells [282]. Taking into account the inverse nature of SIRT6 regulation on LD formation and the protective role of SIRT6 on DNA/chromatin integrity, sirtuins (histone deacetylases) could play a critical role in balancing LD abundance. However, this could also limit the cellular “lipid mass”, including LDs, serving as substrate for lipid-peroxidation-derived genotoxic effects [283] under pro-oxidant conditions. Summarizing, these findings account for a distinct role of cytosolic and nuclear LDs in chromatin and genome surveillance with a particular impact on the aging process, for which the underlying mechanisms remain to be elucidated by further investigation. 

### 4.6. Intracellular Lipid Droplet Trafficking 

Finally, additional findings point to intracellular LD trafficking as an important further aspect in LD biology. In *Drosophila*, LD transport is mediated by the interaction of the motor proteins kinesin and dynein with microtubules [284,285,286,287]. Involved in the coordination of LD movement conferred by cytoskeletal interaction are proteins such as *Bicaudal D* (interacting with dynein) [288] and perilipin-homolog LSD-2 [289], which physically interacts with the gene product *klarsicht* (Klar is identical to the *Drosophila* gene *marbles*) [286,290]. *Klarsicht* mutants develop more or less normally, but due to disturbed LD transport show a markedly reduced lipid/LD deposition in the blastoderm, yielding enhanced transparency of the embryo (hence the name of the mutant). In addition, absence of Klar also leads to mispositioning of photoreceptor nuclei in the developing eye and affects trafficking of secretory vesicles in the salivary gland [291]. Three Klar isoforms (Klar α, β, γ) have been described [292]. While no function is known for Klar γ, the isoform Klar α is involved in linking cytosolic and nucleoplasmic proteins and, as a result, affects positioning of photoreceptor nuclei. Although not shown, it cannot be excluded that Klar α and LD-associated Klar β together with LD-resident Jabba participate in the recruitment of extranuclear histones to LDs during early *Drosophila* embryogenesis, as discussed above. Klar β, in a Klar α-analogous fashion, targets LDs for cytoskeletal interaction, which is mediated by a distinct, C-terminal LD domain [292]. It is noteworthy that Klar via its LD domain serves intracellular, microtubule-based transport of LDs, not only in embryonic but also in adult *Drosophila* tissue, as well as in cultured insect S2 cells [293]. Summarizing, this indicates that the association between LDs and the cytoskeletal/microtubule network may enable distinct intracellular LD positioning mechanics in developmental, physiological, and pathological contexts. 

Taken together, *Drosophila* is a model organism with outstanding findings for LD biology that foreshadow the integrative functionality of LDs in higher organisms connecting environmental, dietary, and hormonal inputs, and assisting in their translation to metabolism and signaling cascades. The interaction with other cellular organelles, primarily mitochondria and the ER, make LDs a useful and highly dynamic organelle apart from lipid storage. However, it has to be admitted that the complexity of the LD interaction network increases with the number of different cell types and tissues, although common central regulatory pathways are conserved from yeast to the mammalian system (Figure 2). In the following chapter dedicated to mammals, we show that LDs, as a kind of “Janus-faced organelle”, essentially fulfill a cytoprotective function, but contribute to age-related diseases if LD accumulation becomes inadequately excessive. 

## 5. LDs in Human Disease

### 5.1. Caloric Restriction, Lifespan Control, and Age-Related Disease 

It is a common thread in evolution that caloric restriction promotes health and prolongs lifespan, which has been documented for a variety of organisms such as *S. cerevisiae*, protozoans, rotifers, crustaceans, *C. elegans* and nematodes in general, *Drosophila melanogaster*, and fish (*Lebistes reticulates* and *Danio rerio*) [32,105,108,294,295,296,297,298,299]. The underlying molecular mechanisms seem to be similar or identical in all the organisms studied and converge to common pathways—TGF-β signaling, IIS/IGF-1 signaling, mTOR, and stimulation of autophagy—as illustrated in Figure 2. As outlined in this figure, these processes also share the common motive that they stimulate or are otherwise associated with LD biosynthesis. Aging research has identified several compounds that preserve health and extend lifespan in different model organisms: resveratrol [83], rapamycin [82], spermidine [212], 2-deoxy-D-glucose [300], curcumin [301], quercetin [83], metformin [302], and NAD^+^ precursors [303]. Many if not all of these substances have been shown to be caloric-restriction mimetics [304] and, as discussed in the previous sections, many of these substances also stimulate LD biogenesis. This provokes the central question of whether the life-prolonging effects of caloric-restriction mimetics observed in non-mammalian animal model systems also apply to mammals and humans, and whether these processes are also LD-driven. 

In rodents, the life-prolonging effects of caloric restriction have been known for a long time [104,305,306]. Over the last few years, data on the life-prolonging effect of caloric restriction also became available for non-human primates, which now allows conclusions to be drawn for humans. Since the 1980s, the effects of caloric restriction have been investigated in the rhesus monkey *Macaca mulatta* by three different organizations (University of Maryland, University of Wisconsin Madison, and the National Institute on Aging). After settling some controversy over the study design, the participating organizations agreed in concluding that caloric restriction has a positive effect on survival and aging-associated diseases [106]. Since these studies did not investigate the involvement of LDs in the life-prolonging effect seen in the rhesus monkeys, a distinct role of LDs in lifespan control can only be conjectured from observations made in other model organisms. Indeed, various findings in mammals, including humans, suggest a positive effect of caloric restriction on LD biology. In humans, a distinction can be made between white, brown, and beige adipose tissue. For a long time it was considered that brown adipose tissue is present only in the newborn, contributing to the regulation of body temperature, and that it is rapidly lost after birth. More recently, however, brown adipose tissue was also found in adults [307], being involved in lipid and glucose oxidation as well as insulin-independent glucose uptake [308]. Based on this, it could be shown that activity of brown adipose tissue increases during adolescence and rapidly ceases at higher age [309]. The implication of brown adipose tissue in the aging process is also supported by the finding that the activity of brown adipose tissue is significantly higher in long-lived than in short-lived animals [308]. Importantly, the influence of caloric restriction leads to the “browning” of white adipose tissue, meaning that adipocytes, instead of forming one large lipid droplet (as in white adipose tissue), constitute many small LDs in brown adipose tissue [310]. This is accompanied by distinct changes in the LD proteome [311]. Due to the long life of primates (in rhesus monkeys, between 30–40 years), the study on caloric restriction referenced above is the only one of its kind hitherto that proves the influence of an anti-aging strategy on primates/humans. Since no data are available at present referring to LDs at the organismic level in aged individuals, we set the focus of this chapter to diseases whose prevalence and severity generally increases with age, and are hence widely accepted as being age-associated [312], and for which, in many cases, a contribution of LDs to the disease pattern have been reported. 

In contrast to the previously discussed models such as *C. elegans* and *D. melanogaster*, the role of LDs in human and mammalian aging is poorly defined. Representing a general conceptual flaw for most pathologies that show elevated cellular LD levels, it is not clear at present whether this LD accumulation is causal to the diseased state or rather is a consequence of disease-related changes in lipid metabolism. In particular, evidence for a regulatory, cytoprotective effect of increased LD levels, such as that indicated by the lifespan-extending effects seen in other, less-complex model systems, is widely missing in the mammalian/human system. Nevertheless, the research listed in Table 1 provides clear evidence that LD levels increase in several, if not all, age-related diseases (ARDs). In ARDs, the irreversible cessation of cell proliferation that demarcates the progression to cellular senescence is discussed as a major pathological criterion [313]. Essential to this, the deregulation of cell-cycle-regulating genes is considered a hallmark of cellular senescence which, surprisingly, also applies to lipid-related pathways [314]. Evidence is accumulating that the deregulation of nutrient-sensing pathways, such as growth hormone and IIS pathway [315,316,317], autophagy–regulatory mTOR and AMPK signaling, and the histone deacetylase activity of sirtuins, play key roles in ARD development [318]. As discussed above, exactly the same pathways have been shown to be important stimuli of LD biogenesis in the other animal models of aging. Insulin signaling is also intrinsically tied to trafficking and storage of lipids in lipid droplets. With respect to the wide range of ADR-associated processes, this review focuses on aging-associated aspects of LD biology in several selected tissues. 

### 5.2. Bone Marrow Aging–Epigenetic Mechanisms

In both children and adults, bone mineral density is inversely correlated with bone marrow fat abundance [350,351] and the aging process in bone marrow is characterized by an expansion in marrow adipose tissue (MAT), which impairs bone stability, thereby yielding an enhanced bone fracture risk [352]. Essential to this, non-differentiated bone marrow stromal cells (BMSCs), amongst others, serve as progenitors for osteoblast and adipocyte differentiation. BMSCs are marked by a low LD content but LD abundance increases upon osteogenesis due to enhanced energy demands, and the blockade of LD formation by Triacsin C (an inhibitor of fatty acyl CoA synthase) results in impaired osteogenic differentiation [353]. In line with this, LD-associated PLIN2 is generally expressed in bone tissue with the highest levels seen in osteo-progenitor cells [353]. Increased serum levels of fatty acids stimulate the adipocytic differentiation of bone marrow progenitor cells [354], the MAT-resident adipocytes containing large LDs that serve as fatty acid and adipokine reservoirs [355]. Of special relevance, MAT expansion may promote free-fatty-acid-based lipotoxicity and negatively affect bone marrow osteoblast proliferation [356], and the inhibition of adipocyte-derived fatty acid synthesis may protect osteoblasts from the lipotoxic effect [357]. 

Epigenetic mechanisms may play a crucial role in bone marrow aging. Underlying this, histone deacetylases (HDACs) such as sirtuins remove acetyl groups from lysine residues of histone tails, which leads to chromatin condensation and thereby alters gene expression. For example, activation of the murine class III HDAC sirtuin 1 (SIRT1), a nutritional sensor responding to NAD^+^/NADH changes, directs mouse mesenchymal C3H10T1/2 cell lines and primary rat bone marrow stromal cells towards enhanced osteoblastic and reduced adipocytic differentiation, while SIRT1 inhibition shows exactly the opposite effect [358]. It is also worth mentioning that LD-derived mono-unsaturated fatty acids are strong activators of SIRT1 [359]. Moreover, a direct interaction has been shown between the peroxisome proliferator-activated receptor γ2 (PPARγ2), a key transcription factor for the differentiation of progenitors into adipocytes, and Sirt1 through its catalytic core domain, forming a stable transcription-inhibiting complex. Binding of this complex to the SIRT1 promoter represses SIRT1 transcription via a self-regulatory feedback loop [360]. This report shows a decline in SIRT1 mRNA and protein levels in older compared to younger human lung, heart, and fat tissues, thus indicating that the SIRT1/PPARγ interaction is a senescence-associated (epi)genetic mechanism [360]. In accordance with this, elevated PPARγ2 levels were also detected in aged compared to young bone marrow stromal cells [361] and it is proposed that the SIRT1/PPARγ2 negative feedback loop lowers SIRT1 expression in bone marrow, and, as a result, promotes MAT expansion and the aging process.

In addition, studies in mice have shown that deletion of another HDAC—Hdac3—leads to lipid accumulation in osteochondrocyte progenitor cells and promotes MAT expansion in young mice [337]. Compared to the wild type, the Hdac3 knockout also leads to a substantial shift in LD/lipid storage-associated Plin1 and Fsp27/Cidec (Fspe27, with fat-specific protein 27 belonging to the family of death-inducing DFF45-like effectors (CIDE) [362]), and a minor but still significant increase in lipolysis-associated lipases (Pnpla2 and Lipe). Further transcriptome analysis in Hdac3 knockout mice revealed a highly abundant expression of 11b-hydroxysteroid dehydrogenase type 1 (Hsd11b1), a gene encoding an enzyme involved in the activation of intracellular glucocorticoids participating in glucocorticoid receptor-based signaling. Inhibition of Hsd11b1 by carbenoxolone resulted in reduced expression of Plin1 and Cidec in Hdac3-deficient cells, which identifies Hdac3 as a crucial regulator of glucocorticoid-induced LD formation in osteoprogenitor cells [337].

### 5.3. Lipid Droplets in Neurodegeneration 

Neurodegenerative diseases such as Morbus Alzheimer and Morbus Parkinson represent another highly prevalent complex of age-related pathologies. Aging of the central nervous system is associated with progressive myelin degeneration at a reduced myelin renewal [363], overall loss of total brain volume [364], glia activation (microglia and astrocytes) and cilia loss in ependymal cells [365], and changes in neuron morphology leading to neuron dysfunction [366]. Lipid homeostasis is of central importance to the functionality of the nervous system since neuron function is considerably impaired by the accumulation of fatty acids that promotes ER stress, lipotoxicity, and mitochondrial damage (recently reviewed in [319]). Notably, the lipid composition of the normal human brain is about 60% by dry weight, which ranks directly after adipose tissue in terms of the tissues with the highest fat fraction, and the brain fat content varies markedly between different brain areas, being highest in myelin (78–81% of the dry weight) and lowest in grey matter (36–40%) [367]. Triacylglycerol levels are low in neurons, probably due to the constant lipid turnover generating the phospholipid mass required for cell membrane maintenance [368]. Although only sparse evidence exists for the in vivo LD formation in neurons, the LD content of neurons increases under “lipid stress” arising from fatty acid treatment [369,370]. In addition, LD numbers are raised upon expression of mutant huntingtin protein [371]. In the aged brain, LD accumulation has been shown in neurons, microglia, astrocytes, and ependymal cells [372,373]. ROS-induced LPO, considered a hallmark of neurodegeneration, serves as a driving force since, similar to the case in *Drosophila*, LPO caused by ROS originating from mitochondrial dysfunction stimulates LD biogenesis by JNK-mediated responses, which precedes the neurodegenerative process in the mammalian system [46]. Astrocytes are important regulators of oxidative stress adaptation in the brain by providing homeostatic, antioxidant support for neurons. This macroglial cell type shows an enhanced resistance towards oxidative stress regimens [374], which might be attributable to its well-equipped antioxidant defenses such as the glutathione redox system, manganese superoxide dismutase, and catalase [375]. Intriguingly, it has been shown that peroxidized lipids (Lox) formed by ROS/LPO in neurons bind to apolipoproteins (especially ApoE) and these ApoE/Lox complexes can be shuttled from neurons to astrocytes, where they are decomposed by lysosomal processing and the liberated fatty acids are stored transiently in LDs [370]. Terminally, the “imported”, LD-bound Lox are oxidized in mitochondria via β-oxidation, which stimulates ROS formation; however, due to the enhanced antioxidant defense, astrocytes may successfully prevent substantial ROS build-up. In good accordance with the findings of Schroeter et al. [375], Ioannu et al. [370] identified the up-regulated expression of several genes associated with antioxidant defenses (Gpx8, glutathione peroxidase 8; superoxide dismutase Sod1 and Sod3; catalase and fatty acid transporters FabP5 and FabP7) in cultured, LD-rich astrocytes. This emphasizes the integration of LDs in enhanced astrocytic antioxidant defenses that confer particular antioxidant robustness needed in these macroglial cells to serve as a “detoxifying recipient” for neuron-derived LPO products. Hence, it appears likely that disturbances of this neuron-to-astrocyte lipid transfer and LD storage detoxification mechanism will enhance the risk of neuron damage and the development of Alzheimer’s disease (AD). In humans, three APO-E alleles have been described: APOE2, APOE3, and APOE4. Compared to APOE2 homozygotes, individuals bearing the APOE4 allele either in a heterozygous or homozygous genotype show a 9–15-fold increased risk of acquiring AD, rendering the APOE4 allele as one of the main risk factors for AD [376,377]. In fact, it was shown that the APOE4 allele dampens the “neuron to glia” lipid transfer, thus promoting neurodegeneration [378]. With respect to this, it is tempting to speculate that, downstream of ApoE4/Lox-shuttling, the inability of astrocytes to store Lox/lipid peroxides in LDs also promotes the risk of developing AD. 

LDs and α-synuclein. In Parkinson’s disease (PD), the second most common neurodegenerative disease after AD, LDs also move into the focus of research. Central to PD pathogenesis is the loss of dopaminergic neurons in the substantia nigra pars compacta accompanied by the appearance of Lewy bodies [379]. Lewy bodies are insoluble aggregates of misfolded proteins, of which α-synuclein comprises the main constituent [379,380]. Oligomers of α-synuclein are neurotoxic to themselves and are considered as the main drivers of neurodegeneration [381], with mutations of α-synuclein dramatically increasing the prevalence of PD [382]. It was shown that α-synuclein forms di- and trimers that accumulate at the surface of LDs in human embryonic kidney cells as well as hippocampal neurons treated with high concentrations of oleic acid [325]. Taking into account the observations made in *S. cerevisiae, C. elegans*, and *Drosophila*, this suggests the existence of LD-based detoxification mechanisms for α-synuclein aggregates in higher organisms, which hypothetically may provide neuroprotection. Whether this holds true and the extent to which it may play a role in PD pathogenesis remains to be addressed by further research. 

LD accumulation in Huntington’s disease. Apart from AD and PD, accumulation of LDs is seen in several other neurological and neurodegenerative disorders, including Huntington’s disease and amyotrophic lateral sclerosis (ALS), as reviewed recently by Islyme et al. [383]. However, the authors also leave open the question of whether LD accumulation mitigates or promotes the progression of neurodegenerative disorders. This emphasizes the ambiguity of LD biogenesis in the pathogenic context. LD accumulation may enable dynamic lipid storage, and, as a result, act as a potential substrate for LPO, in one pathological condition, but opposite to this, in another diseased state, resemble a sink for the “safe” sequestration of LPO-derived compounds including peroxidized fatty acids as well as aggregates of potentially harmful proteins such as α-synuclein oligomers. It is reasonable to consider that the direction of the LD-based response is dependent on the influence of additional factors. For instance, in Huntington’s disease, the mutation-based excessive N-terminal poly-glutamylation of the protein huntingtin (polyG-Htt) is causative of neuronal cell death, which to a substantial degree is due to the disturbed interaction between poly-glutamylated huntingtin and the cytoskeleton [384]. Huntingtin is involved in several cellular transport processes and, in concert with the microtubule network in particular, participates in the axonal transport of organelles and neurotransmitters in neurons [385]. Interestingly, in a yeast model of poly-glutamylation, polyG-Htt aggregation leads to the formation of inclusion bodies and cell death, the degree of which correlates with an aberrant LD morphology that is indicative of a disturbed TAG storage function [386]. Taking into consideration the role of cytoskeletal alterations in neurodegenerative disease [387], an inadequate binding of protein aggregates to LDs could hypothetically also result in a disturbance of cytoskeleton-based intracellular LD/lipid trafficking, for instance, by impairing the afore-mentioned interaction of LDs with motor proteins (see Section 4), and, as a result, aggravate disease progression. Therefore, although the clearance of potentially dangerous protein aggregates by LDs may be considered beneficial to cell integrity, evaluating the impact of this LD-based mechanism on disease outcome deserves a rather holistic approach integrating a multiplicity of interlinked accessory factors.

### 5.4. Lipid Droplets in Metabolic Disease

Intimately associated with the essential, lipid-metabolism-linked function of LDs, the metabolic process itself represents a critical element of aging. This is indicated by the fact that the risk for metabolic diseases such as the metabolic syndrome, type II diabetes, non-alcoholic fatty liver disease/steatohepatitis (NASH), and cardiovascular diseases and atherosclerosis, is increasing with age [388]. For example, in the pathogenesis of type II diabetes, the development of insulin resistance represents an early, critical issue leading to the disturbance of glucose homeostasis. As a pathophysiological response, insulin production by the β-cells of the pancreatic Langerhans islets becomes elevated, which partially compensates for the incremental insulin resistance. However, with progression of the diabetic condition, the β-cell mass declines and, with this, insulin production ceases. Nutrition-derived lipid stress arising from a high-fat diet is under discussion as a main driver of type II diabetes development, with LDs playing a central role in β-cell lipid management [342]. In fact, it was shown that knockdown of perilipin 2 (PLIN2), an essential LD scaffold protein, resulted in reduced insulin production by β-cells, whereas PLIN2 overexpression boosted insulin secretion. Moreover, β-cells devoid of LDs are prone to ER stress, which results in an impairment of β-cell functionality [343]. This accounts for a critical role of LDs in protecting β-cells from the toxic effects of lipids, and, as a result, acting as an antagonist of type II diabetes progression.

LD accumulation in NASH. Insulin resistance is closely associated with obesity marked by the enhanced accumulation of LDs in epithelial cells and other non-adipose tissues, which establishes a pro-inflammatory microenvironment in the affected tissues as commonly found in metabolic diseases. This also applies to the development of NASH, the inflammatory type of non-alcoholic fatty liver disease (NAFLD) [389]. NASH, which is closely associated with insulin resistance, represents a well-studied liver pathology emerging from chronic fat-rich alimentation. NASH is characterized by the marked accumulation of triglycerides in liver epithelial cells (i.e., parenchymal hepatocytes) causing liver steatosis and the accompanying, chronic inflammation that promotes fibrotic/cirrhotic remodeling of liver tissue (recently reviewed in [390]). Of particular relevance, the development of NASH is accompanied by several processes including mitochondrial dysfunction, ER stress, and enhanced ROS formation, as well as tissue-specific changes, primarily the activation of hepatic stellate cells (HSCs), which promotes the inflammatory process and liver fibrosis upon trans-differentiation of activated HSCs into the extracellular matrix (ECM) producing myofibroblast-like cells [344]. In the “*multiple hit pathogenesis*” of NASH, toxic lipids play an essential role, affecting different liver cell populations, in particular parenchymal hepatocytes, HSCs/myofibroblast-like cells, and *Kupffer* cells (i.e., liver macrophages) in different ways [391]. Contrasting with the well-defined general understanding of NASH development and its clinical manifestation, little is known about age-associated alterations in lipid metabolism and LD biogenesis in NAFLD/NASH progression. Nevertheless, ROS and the ROS-mediated senescence of hepatic cells may also play a pivotal role in NAFLD/NASH pathogenesis. For instance, it was shown that liver steatosis is promoted by a decline in mitochondrial fatty acid metabolism in senescent hepatocytes, an effect that was abolished by the antioxidant, lipid peroxidation inhibitory flavonoid quercetin [217,392]. 

Role of ROS. Several investigations account for a distinct role of the ECM-associated matrix protein CCN1 (central communication network factor 1; formerly termed Cyr61, cysteine-rich protein 61 [393]) in NASH progression. CCN1 plays an important role in wound-repair-associated ECM remodeling via binding to integrin α_V_β3 and α_V_β5 of epithelial cells and myofibroblast integrin α6β1 [394]. In line with this and accounting for a role in liver fibrosis, an increased expression of CCN1 has been shown in the liver of NASH patients [395], in hepatocytes of the human cirrhotic liver, and as a reaction to liver injury [396]. Furthermore, it has been shown that CCN1 stimulates hepatic steatosis in obese mice and promotes LD accumulation in hepatocytes treated with free fatty acids [395]. Of considerable relevance, CCN1 expression is enhanced by ROS. This was demonstrated in skin fibroblasts exposed to hydrogen peroxide, which resulted in the c-jun/AP1-dependent up-regulation of CCN1 expression yielding a repression of collagen synthesis and fibroblast senescence [397,398]. Similarly, the overexpression of CCN1 also lowers the production of collagen type1α1 (col1α1) in HSCs [399]. Moreover, CCN1, by acting via integrin α6β1, shifts ROS/RAC1-dependent NOX1 (NADPH oxidase 1) activity and promotes senescence of HSCs as well as myofibroblasts, as a result conferring an anti-fibrotic response, and in addition stimulates liver-regeneration-associated signaling via IL-6 (interleukin-6) and CXCR2 (chemokine receptor 2) ligands [396,400]. 

These observations suggest a seemingly ambiguous involvement of CCN1 in NASH: promoting steatosis by LD accumulation in parenchymal hepatocytes, but counteracting NASH-associated fibrosis by mediating senescence, in non-parenchymal HSCs and myofibroblasts. It is noteworthy that in HSCs, the overexpression of CCN1 is also able to trigger ER stress and UPR due to the high abundance of CCN1 protein, which renders these cells susceptible to apoptotic cell death [399]. Hence, elevated CCN1 levels may exert a particular challenge for cell integrity in HSCs. Importantly, parenchymal hepatocytes, and not HSCs or Kupffer cells, represent the major hepatic source of CCN1. This was shown by employing CCl_4_, a hepatotoxic compound that generates free radicals upon cytochrome P450-based metabolization and shifts CCN1 expression only in parenchymal hepatocytes [396]. It has to be mentioned that CCN1 null mutant mice reveal a normal hepatic function in the absence of CCl_4_, which suggests a specific association of hepatocytic CCN1 expression with stress/ROS-mediated conditions, such as existing in liver injury and inflammation. This notion is supported by the further observation that CCN1 expression in parenchymal hepatocytes is also up-regulated by the pro-inflammatory cytokine TNFα in a ROS-dependent mode [401]. Conversely, the before-mentioned stimulation of LD biogenesis upon CCN1 overexpression in primary mouse hepatocytes is accompanied by an enhanced expression of TNFα [42]. TNFα, acting via the TNFα receptor (TNFR), stimulates mitochondrial ROS generation via JNK signaling and downstream ER stress, which leads to the activation of ATF6 (activating transcription factor 6) and elF2α (eukaryotic initiation factor 2α). In turn, ATF6 and elF2α transduce the signal to nuclear transcriptional control via C/EBP (CCAAT/enhancer-binding protein α) homologous protein (CHOP), which may stimulate ER-stress-dependent apoptosis in hepatocytes [402].

Taken together, these findings account for the existence of an autocrine amplification loop in hepatocytes, established by CCN1 and TNFα/TNFR under pro-oxidant conditions that enables the secretion of CCN1 to the extracellular space and drives activated HSCs and myofibroblasts towards senescence, eventually counteracting the HSC/myofibroblast-driven fibrotic process. Indeed, an inflammation-associated feed-forward loop of cytokine secretion including TNFα has been discussed recently [402]. In extension to this, CCN1-stimulated LD biogenesis could play a central, albeit ambiguous, role in this regulatory network. With respect to the causative role of increased CCN1 and TNFα levels in ER stress, UPR, and apoptosis in HSCs, as well as hepatocytes, it is appropriate to consider that hepatocytes, as the main hepatic source for CCN1 under stress conditions, need to be protected from the cytotoxic potential established by the self-amplifying CCN1–TNFα circuit. With respect to this, the finding of Ju et al. revealed that overexpression of CCN1 in hepatocytes leads to both (i) the up-regulation of lipid-metabolism-associated genes (ii) and the up-regulation of sirtuins (Sirt 1, 2 and 3), Nrf1, BMP2 (a member of the TGF-β superfamily), and AMP kinases [395]. This connects the CCN1–TNFα circuit to hepatic LD biogenesis, and by this steatosis to most of the LD-associated signaling pathways discussed above, which are capable of exerting a cytoprotective function in cells under oxidative stress. Hence, it is tempting to speculate that enhanced LD biogenesis allows hepatocytes to synthesize CCN1 in potentially cytotoxic amounts, which are needed for counteracting inflammation-driven fibrosis by the paracrine induction of HCS/myofibroblast senescence. Obviously, if this holds true, such a mechanism would assign a yet ambiguous context to LD biogenesis in NAFLD/NASH (as well as alcoholic AFLD): slowing disease progression in a paracrine mode via the CCN1-mediated deceleration of inflammation-associated fibrosis at the cost of promoting steatosis progression via an autocrine amplification loop. 

The stimulation of NOX1-based ROS (superoxide) production following CCN1 signaling via integrinα6β1 in HSCs represents a further critical element of such auto- and paracrine regulatory networks. Concerning this, Kim et al. proposed that HSC senescence is caused by CCN1/NOX1-mediated ROS leading to genotoxic damage, and p53 and p16/pRb–dependent, senescence-related responses [396]. Moreover, similar to LD accumulation mediated by CCN1 in hepatocytes [395], Long et al. showed that the up-regulation of NOX1 also stimulates lipid-metabolism-related gene expression and LD accumulation in mouse hepatocytes. and also leads to the up-regulation of ER-stress-associated genes ATF6 and eIF2, effects which were antagonized by the antioxidant N-acetylcysteine (NAC) [403]. This strengthens the assumptions made above regarding a protective role of LD biogenesis counteracting the pro-oxidant effects on the CCN1–TNFα circuit, as well as altered NOX1 activity in hepatocytes under conditions of inflammation. It is noteworthy to emphasize that in the experiments conducted by Long et al., NOX1 overexpression was accomplished in hepatocytes via knockdown of the transcription factor hepatocyte nuclear factor 1β(HNF1β), which identifies HNF1β as a negative regulator of NOX1, and, as a result, a suppressor of both NOX1-mediated superoxide formation and LD biogenesis in hepatocytes [403]. Interestingly, steatotic livers of obese mice show a reduced expression of HNF1β, and treatment of mouse hepatocytes with palmitic acid also lowers HNF1β expression, an effect that is suppressed by NAC [403]. In addition, the HNF1β knockdown also stimulated insulin resistance in hepatocytes, which was also ameliorated by NAC. These observations account for a further feedback mechanism in hepatocytes, leading to an elevated LD accumulation that is driven by the lipid/ROS-based down-regulation of HNF1β, and resulting in NOX1-mediated LD biogenesis and causing increased insulin resistance. With respect to this, HNF1β connects NAFLD with diabetes, which is underlined by the finding that mutated HNF1β alleles are associated with diabetes type MODY5 (maturity-onset diabetes of the young) [404] as well as type-II diabetes [405], suggesting that HNF1β activity mitigates insulin resistance, at least in these pathologies.

Lipid droplets, lipophagy, and hepatic lipid homeostasis. The hepatic lipid flux is marked by diurnal oscillations of fed and fasted states reflected by LD catabolism (fed state) and LD storage (fasted state), with the hepatocyte LD lifecycle playing a pivotal role in systemic lipid homeostasis. Regarding this, the blood insulin concentration is critical by linking systemic fed/fasted states to oscillating high (fed) and low (fasted) insulin signaling. Among several physiologic contexts, this nutrition-dependent systemic insulin dynamics also affects cellular LD dynamics, in particular intracellular LD trafficking. Similar to the case of *Drosophila* (see Section 4), LDs may also become connected to the cytoskeleton in primary hepatocytes where LD binding of the motor protein kinesin-1 mediates their transport along microtubules, a process that is regulated by insulin signaling [406] and serves the delivery of LDs to the smooth ER (sER) for VLDL (very-low-density lipoprotein) production [407]. Essential to this, insulin signaling enhances the GDP-dependent binding of GTPase ADP-ribosylation factor 1 (ARF1) to LDs, rendering them “reactive” [408], the bound ARF1 in turn recruiting phospholipase-D1 (PLD1), which generates phosphatic acid (PA), and, as a result, shifts the PA content of reactive LDs [409]. As a consequence, via binding to PA, these LDs recruit kinesin-1, which terminally mediates LD shuttling to the sER. Therefore, in the fed state, high systemic insulin levels will promote LD–sER shuttling and fuel VLDL production as well as secretion by hepatocytes, while the postprandial, low-blood insulin levels characteristic of the fasted state will antagonize the shuttling process and thus limit VLDL synthesis and secretion. Notably, starvation conditions markedly enhance the hepatic clearance of adipose-tissue-derived lipids from the circulation, which causes a substantial shift in the hepatocyte LD content. Hence, the down-regulation of LD trafficking entailed by low insulin signaling serves as a “bottleneck” for VLDL production in LD-rich hepatocytes, limiting VLDL under fasted conditions. This puts emphasis on the role of LDs participating in the physiological regulation of systemic lipid homeostasis serving as a hormonally controlled, dynamic lipid buffer in the liver. 

At the cellular level, LDs can be selectively degraded by autophagy, a process termed (macro)lipophagy, which sequesters cytosolic LDs for autophagolysosomal digest and is pivotal to lipid metabolism [410,411]. In addition to its role in mobilizing fatty acids from cellular LD-based lipid stores, lipophagy represents a pivotal “guardian” of cellular LD abundance. This holds particularly true when LD biogenesis is substantially stimulated in hepatocytes in response to an enhanced clearance of lipids from the blood stream when facing the risk of systemic lipotoxicity arising from an excess of circulating free fatty acids. Emphasis on this is provided by experiments showing that lipid treatment of cultured hepatocytes stimulates lipophagy while lipophagy inhibition by the macroautophagy inhibitor 3-methyladenine shifts the number of LDs in hepatocytes even under normal culture conditions [410]. Moreover, lipophagy is also enhanced in the mouse liver under starvation conditions that stimulate LD biogenesis. Importantly, these experiments revealed further that enhanced exogenous lipid supply such as that provoked by a fat-rich diet negatively affects LD breakdown by lipophagy. In good agreement with this, it was reported recently for a mouse model of obesity that lipophagy declines upon feeding a high-fat diet, resulting in liver steatosis [412]. Of special relevance to lipotoxic side effects, the observed drop in autophagic/lipophagic efficiency was accompanied by the enhanced accumulation of HNE-modified proteins. This connects lipophagy with reparative autophagy (i.e., detoxification of the aggregated modified proteins), and, as a result, LD abundance, with the critical interference existing between LPO and proteostasis. Hence, it is not surprising that a reduced lipophagic flux plays an important pathogenic role in NAFLD and other lipid-metabolism-associated diseases such as atherosclerosis [413], and may affect the aging-associated transition from NAFLD into primary hepatocellular carcinoma (HCC) [344]. 

LD accumulation, oxidative stress, and cell death. Finally, the excessive cytosolic accumulation of fatty acids seen under “hyperlipidemic” states such as obesity, NAFLD/NASH, and diabetes may lead to an elevated susceptibility to lipotoxicity-induced cell death via apoptosis. The specific term lipoapoptosis was coined for apoptotic cell death stimulated by fatty acid derivatives such as ceramide [414], and as a terminal issue in lipotoxic settings, lipoapoptosis is of particular relevance to several lipid-associated pathologies including NAFLD [415], as well as others such as vascular and cardio-metabolic diseases [416], which are discussed below. In addition, ROS-stimulated LPO yields further metabolites such as HNE, which interferes with anti-apoptotic and cell-proliferation-associated intracellular signaling [417], but is also capable of inducing apoptosis per se [418]. Moreover, the concept of ferroptosis, which has gained substantial interest over the last decade, also represents, in principle, an LPO-dependent mode of cell death. In ferroptosis, LPO is initiated by iron-derived ^•^OH radicals at inadequate antioxidant defenses (i.e., weakening of the GSH/GSSG redox system due to GSH-peroxidase 4 deficiency) leading to a non-apoptotic, necrotic mode of cell death [419]. Taking into consideration that hepatocytes represent an iron-rich cell type serving systemic iron buffering, ferroptosis represents a considerable issue in NAFLD/NASH that also occurs with high levels of PUFAs [420]. In addition, it should not be overlooked that ferroptosis may not only lead to hepatocyte loss, but as a necrotic mode of cell death will also aggravate the pro-inflammatory condition. Therefore, lipid stress arising from an inappropriate lipid accumulation will affect many cellular targets and pathways, among which LDs are of central relevance, either fueling lipid (per)oxidation or attenuating lipotoxicity by aiding cellular lipid detoxification and interfering with cytotoxic responses such as lipoapoptosis and possibly also other lethal outcomes such as ferroptosis. 

### 5.5. Lipid Droplets in Vascular Disease 

In hepatic Kupffer cells residing in the liver sinusoids, the augmented uptake of cholesterol and free fatty acids enhances the development of a lipid-rich macrophage phenotype [421], characterized as foam cells, and the aggregation of such lipid-rich Kupffer cells contributes to NAFLD/NASH-associated lipogranulomas that are built from inflammatory cells, ECM (collagen), and LDs [422,423]. Of particular pathological relevance, the conversion of macrophages into foam cells represents a major issue in atherosclerosis development (atherogenesis), since the “foamy” macrophages build up atherosclerotic plaques in arterial walls, leading to inflammation and progressive damage to the vessel wall (reviewed in [328]). Underlying this is the oxidation of low-density lipoprotein (LDL) bound to proteoglycans of the extracellular matrix and the vessel endothelium, which yields oxidized LDL (oxLDL), the oxLDL in turn triggering the release of monocyte chemoattractant protein (MCP-1) by vascular endothelial cells and vascular smooth muscle cells (VSMCs). The attracted monocytes migrate to the arterial wall and differentiate into macrophages that endocytose the oxLDL particles via scavenger receptor A (SR-A) and CD36 [424], although alternative uptake mechanisms may exist [425]. Upon lysosomal processing of the oxLDL particles, the free fatty acids and cholesterol molecules are released to the cytoplasm, where they are either stored in LDs [426,427] or are released via high-density lipoprotein (HDL) [428]. The enhanced uptake of oxLDL by macrophages will nourish the accumulation of cholesterol-rich LDs, and, as a result, stimulate foam cell and plaque formation [328]. It should not be overlooked that LDs are also essential to reverse cholesterol transport (RCT), a process by which cholesterol sequestered from the circulation is stored transiently in LDs, from which it can be liberated via lipophagy and be re-released from the cell, for instance, to the bile for fecal excretion. Although RCT can be accomplished by several cell types, macrophage-associated RCT is considered causal to the atherosclerotic process [429]. 

LDs and cholesterol homeostasis. From this, it becomes clear that the ability of LDs to store cholesterol is essential to cellular cholesterol homeostasis and the protection from cholesterol lipotoxicity. Essential to this is the esterification of free, unesterified cholesterol by Acetyl-coenzyme A cholesterol *O*-acyltransferase-1 (ACAT1) as a prerequisite for the incorporation of the cholesteryl-esters into LDs [430]. In line with the protective role of LDs, stimulation of ACAT1 is essential for proper cellular cholesterol management and, by facilitating LD-based cholesterol clearance, counteracts cholesterol toxicity [431]. In addition, oxysterol-binding protein-related proteins ORP2, ORP5, and ORP8 can stimulate LD biogenesis and can bind oxysterols such as 25-hydroxycholesterol and 7-ketocholesterol, as well as cholesterol itself, to LDs [432,433]. Considering the toxic effects of oxysterols, oxysterol binding to LDs clearly represents a cytoprotective function of LDs. This holds particularly true for 7-ketocholesterol, which accumulates in foam cells (reviewed in [434]) and is known as a stimulator of oxiapoptophagy, a distinct mode of oxysterol/oxidative-stress-associated cell death involving apoptosis and autophagy with particular pathogenic relevance to age-related diseases including atherosclerosis [435,436,437,438,439]. The cholesteryl ester-driven biogenesis of LDs in vascular macrophages is considered causal to foam cell development [429], which likewise also holds true for oxysterol binding to LDs, and both processes represent driving forces of atherogenesis. As stated by Lee-Rueckert et al. [440], the development of foam cells may be accompanied by the reduced expression of pro-inflammatory genes (characteristic of the activated M1 macrophage phenotype) converting the phenotype into an anti-inflammatory one (i.e., activated lipid-rich M2 macrophages [441], leading to the concept that foam cell development represents an anti-atherogenic effect [440]. Hence, LD accumulation in atherosclerosis serves as a further example of the ambiguous role of LDs in pathophysiological settings, as mentioned above for NAFLD: protection from acute lipotoxicity, thus aiding cell survival at the cost of promoting a chronic process such as atherosclerosis and liver fibrosis/cirrhosis. Interestingly, Lathe et al. followed a similar concept in discussing the effects of the pathogen (virus)-induced stimulation of 25-hydroxycholesterol, which via ACAT1 esterification can also bind to LDs, and contributes to both atherosclerosis as well as Alzheimer’s disease, postulating that 25-hydroxycholesterol protects from “*infectious agents at the expense of longer-term pathology*” [442]. Emphasizing the ambivalent role of LD formation in foam cell development and the atherogenic context, plaque formation is accompanied by a decline in the lipophagic flux, which limits LD breakdown, and, as a result, excessive liberation of cholesterol [443], but aggravates atherogenesis due to the continuous stimulation of foam cell formation driven by LD accumulation. Finally, it appears noteworthy that VSMCs may translocate to the arterial intima in the course of atherosclerosis progression and transdifferentiate into a macrophage-like foam cell phenotype, revealing an enhanced oxLDL content, although containing fewer LDs and showing a reduced lipophagic flux compared to macrophage-derived foam cells [444,445]. Nevertheless, VSMC-derived foam cells can comprise about 50% of the foam cell content seen in human atherosclerotic plaques [444], and thus represent an atherogenesis-associated cell population of considerable interest. 

Finally, LDs may assist macrophage integrity not only by the control of lipid balance and oxLDL/oxysterol sequestration, but also by aiding the “clearance” of other aging and stress-associated compounds, especially protein aggregates. This is indicated by a recent investigation that demonstrates the aging-dependent binding of protein aggregates to LDs in mouse intestinal tissue, supposedly followed by the terminal degradation of the critical matter via lipophagy [135]. It is conceivable that a similar, LD-based cytoprotective mechanism is involved in aging and lifespan control of other organisms such as *C. elegans* and *Drosophila*, and also likely in yeast, considering the binding of IBs to LDs as discussed in this review. 

### 5.6. LD Accumulation in Cardiomyocytes: Role of PPARs

As in other tissues, LDs also play a dual role in the cardiac system. To overcome the enhanced energy demand of cardiomyocytes, long-chain fatty acids (LCFAs) such as palmitate and oleate (due to their higher energy yield per carbon molecule as compared to glucose) are the primary fuel needed for ATP synthesis [330]. Subsequent to esterification by acyl-coenzyme A synthetase (CoA), these CoA-fatty acyls are further esterified to a glycerol backbone and stored as TAGs in LDs. Upon LD lipolysis and lipase-mediated TAG hydrolysis, the liberated fatty acids will fuel mitochondrial β-oxidation or serve as ligands for the nuclear peroxisome proliferator-activated receptor α (PPARα), a transcription factor that is central to the control of intracellular TAG turnover and fatty acid metabolism [446,447]. It is noteworthy that another PPAR species, PPARγ, stimulates lipid uptake and LD biogenesis in cardiac tissue and confers protection of cardiomyocytes from ROS-mediated damage via regulating the expression of the Sod2 gene, encoding manganese superoxide dismutase [448]. Hence, PPARs represent important determinants of cardiac LD turnover, which is central to cardiac lipid management and protects the heart from organ dysfunction caused by lipotoxicity [330]. Interestingly, it has been shown that PPARα together with mTOR also regulate a reciprocal mode of LD biogenesis and mTORC1-containing stress granule formation in lipid-stressed HEK239T and SH-SY5Y cells [449]. It cannot be excluded that this also applies to heart tissue and this interconnects cardiac LD turnover with autophagy via mTOR/PPAR signaling.

Role of PLIN5 in cardiac disease. Of particular pathologic relevance, heart failure in obesity and diabetes mellitus is associated with hyperlipidemia resulting in lipid accumulation and an expansion of the myocardial LD content [450]. In diabetic heart disease, particular attention has been paid to the role of the LD-associated protein perilipin 5 (PLIN5) (recently reviewed in [451]. Reflecting the context-dependent role of LDs in lipid homeostasis, PLIN5, under normal conditions, inhibits lipolysis via binding comparative gene identification-58 (CGI-58), which otherwise binds triglyceride lipase, but under stress conditions (e.g. fasting, exercise) stimulates lipolysis as a result of phosphorylation by PKA (protein kinase A), leading to the release of CGI-58 from PLIN5, which in turn activates triglyceride lipase [452]. Connected with this, cells with a high oxidative capacity show an enhanced expression of PLIN5, as holds especially true for cardiomyocytes, where PLIN5 is responsible for the tethering of LDs to mitochondria [453]. Compared to healthy control donors, expression of PLIN5 is reduced in samples drawn from patients with heart failure, showing a decline in direct LD-mitochondria contacts and reduced fatty acid usage for energy supply [454]. On the other hand, by inhibiting triglyceride lipase, PLIN5 supports the sequestration of TAG by LDs, which will limit fatty acid availability, and, as a result, protect the heart from lipotoxicity; however, dysregulation of this process upon PLIN5 overexpression will promote cardiac steatosis and hypertrophy [455,456]. Conversely, LDs are absent from myocardial tissue in PLIN5 knockout mice and myocytes isolated from these PLIN5^−/−^ mice show an increased fatty acid oxidation in vitro compared to the wild type. Moreover, ROS production is enhanced in the heart tissue of PLIN5^−/−^ mice, which aggravates the age-related cardiomyopathy, but can be antagonized by the glutathione-precursor N-acetylcysteine [457]. In addition, PLIN5 may also exert protection from lipotoxicity by antagonizing ER stress, which has been shown for pancreatic β-cells upon chronic exposure to free fatty acids [458]. Finally, a recent report demonstrated a regulatory role for cardiac PLIN5 in cardiac Ca^2+^ signaling and muscle contractility that is based on the interaction between PLIN5 and sarcoplasmic/endoplasmic reticulum Ca^2+^ ATPAase2 [459]. Summarizing, these findings put emphasis on the proper LD balance and expression of LD-associated PLIN5 on heart integrity maintenance. 

### 5.7. Lipid Droplets and Cancer—A General Outline

Growing evidence suggests a manifold involvement in LDs in cancer (reviewed in [460]), however, it still is not clear whether LD accumulation plays a causative role in carcinogenesis (as, for instance, is discussed above for the transition from NAFLD to HCC), or is a consequence of increased lipid demands of tumor cells; or—most likely—both may even apply. In many aspects, aging and tumorigenesis show opposing phenotypes, which led to the proposal that anti-aging strategies can be developed based on tumor cells [461]. Attributable to the altered energy demands of tumor cell proliferation, elevated LD accumulation is observable in different kinds of tumors, such as colorectal cancer, hepatocellular and pancreatic carcinoma, renal cell carcinoma, prostate and breast cancer, lung cancer, and glioblastoma [462,463,464]. For several cancers, a direct correlation between tumor cell survival, tumor aggressiveness, and LD numbers has been documented and tendencies exist to consider cancer as a “LD-driven metabolic disease” [465]. A series of excellent reviews addresses the question of how LDs can promote tumorigenesis [460,462,465], which are recapitulated here briefly. As stated above, the most obvious role of LDs in cancer growth is energy supply, with LD-derived FAs serving as fuel for β-oxidation and mitochondrial ATP production [466]. LD-resident PLIN5 is essential to the FA flux between LDs and mitochondria [453], which is essential to cellular lipid supply coping with increased energy demands such as seen in tumor cells, but also in normal cells under stress conditions as discussed above for cardiovascular disease. Of special pathophysiological relevance, the FAs released from LDs are not only used for energy production, but also act as signaling molecules (e.g., lysophosphatidic acid) regulating tumor progression and metastasis [460,467]. Furthermore, LDs are able to modulate cell cycle checkpoints and gene expression in tumor cells (e.g., G_0_/G_1_ bypass and regulation of FOXO3A activity) [460,468,469]. In addition, LDs also enable the intracellular trafficking of growth-signaling proteins such as PI3K, ERK1, ERK2, p38, and PKC, as well as endo-/transcytosis-regulating caveolin, which are also involved in tumorigenesis [470,471].

Moreover, LDs seem to be especially important for tumor initiation during early carcinogenesis. In the so-called elimination phase, tumor defense by the both the innate and adaptive immune systems is based on the detection of potentially malignant cells and their targeted elimination via apoptosis [472,473]. Besides acting inside the tumor cells, LDs also interfere with the tumor microenvironment [460]. As an illustrative example of the highly complex interactions between LDs, tumor cells, and the tumor cell microenvironment, the role of LDs in cellular eicosanoid production [474] should be mentioned here. Eicosanoids (e.g., prostaglandins, leukotrienes, and lipoxins) are important PUFA-derived (e.g., arachidonic acid) signaling molecules, which are secreted from tumor cells into their microenvironment where they exert autocrine and paracrine activities. For instance, prostaglandin E2 (PGE_2_) is mainly synthesized from LDs in cancer cells [475] and immune suppression conferred by tumor-derived PGE_2_ is deeply involved in the tumor escape from immune surveillance [476]. In a complementary mode, PGE_2_ is also involved in tumor cell proliferation, angiogenesis, and metastasis [477]. Conversely, dendritic cells enriched in LDs containing oxidized TAG show a dysfunctional antigen presentation [478], which will also impair the host tumor defense.

Acting on the central balance of homeostatic growth control, LD accumulation may also affect the onset of apoptosis [479,480], probably by delaying the accumulation of toxic fatty acids inside the affected tissue [481,482]. Several findings account for the direct involvement of LDs in cancer cell apoptosis. Notably, in both tumorous and non-tumorous cell lines, stimulation of apoptosis occurs in conjunction with enhanced LD biogenesis [35,483], and evidence exists that an increased LD content improves the tumor cells’ resistance to pro-apoptotic stimuli. This may be due to the enhanced sequestration of a pro-apoptotic stimulus by LDs, as was demonstrated for curcumin. This plant polyphenol stimulates apoptosis via intrinsic, mitochondria-dependent signaling in several cell lines [484,485,486,487], but fails to do so in glioblastoma cells [488]. In these cells, curcumin is efficiently sequestered by the high LD content. Lowering LD numbers via inhibition of cytosolic phospholipase A2 restores the sensitivity to curcumin-mediated apoptosis [488]. In a similar way, the enhanced sequestration of chemotherapeutic drugs by LDs may render cancer treatment inefficient and finally promote drug resistance [460,489]. 

However, further approaches exist to explain the anti-apoptotic role of LDs, addressing intrinsic, mitochondria-dependent (MOMP/apoptosome), and extrinsic, death-receptor-dependent (TNFα receptor superfamily /DISC) apoptotic signaling [490]. For instance, it was shown that alterations of the cholesterol content of lipid rafts blocks the onset of apoptosis induced upon TRAIL (tumor necrosis factor-related apoptosis-inducing ligand) ligation to death receptors DR4 and DR5 in non-small cell lung carcinoma cells [491]. Considering that LDs serve as a reservoir for cholesterol, LDs could hypothetically contribute to the suppression of extrinsic apoptosis. In addition, our own findings suggest a direct involvement of LDs in intrinsic apoptotic signaling. We showed (for details see Section 2.3) that mitochondria-localized apoptotic proteins (pro- as well as anti-apoptotic) contain a V-domain that enables shuttling of these proteins from mitochondria to LDs. The affinity of this V-domain is higher for LDs than for mitochondria and, upon an increase in the cytosolic LD content (as seen in tumor cells), these apoptotic proteins are cleared from the mitochondria, with the relocalization to LDs interrupting the apoptotic program [35]. In fact, both pro- and anti-apoptotic proteins such as BAX [12,35,492], BCL-X_L_ [35], Bcl-w [12], AIFM1, AIFM2 [12], CCAR2 [12], API5 [492], and TPT1 [35,492] were shown to localize to LDs in tumor cells.

Taken together, it is likely that LDs play a hitherto underestimated role in cancer biology, addressing tumorigenesis at several critical instances. As discussed in this review, LDs may confer protection by exerting antioxidant properties including lipid stress (LPO) under healthy conditions. On the contrary, LDs may promote carcinogenesis in diseased contexts, especially in chronic, inflammation-associated settings such as the “*malignant*” transition from NASH to HCC, and may terminally also contribute to tumor progression and metastasis by interfering with vascularization and proliferation–regulatory cell signaling in the tumor environment.

## 6. Concluding Remarks

In most model organisms, a clear picture seems to emerge that LDs, despite their rather negative appraisal as a mere “fat-particle”, fulfill a cytoprotective role. This is due to the “buffering” function of LDs, which enables them to take up lipid peroxides and other oxidized lipid derivatives (e.g., oxLDL), as well as to detoxify misfolded proteins and protein aggregates in and on various cell organelles. Accordingly, it is not surprising that lifespan extension is positively correlated with LD abundance (at least to some extent). Similar experimental evidence can be found throughout a diversity of biological model systems, suggesting LDs inherit highly conserved functions. This picture is clearest in simple organisms such as *S. cerevisiae* or *C. elegans*, but is also presented by the more complex organism *D. melanogaster*. Central to this LD–aging connection seems to be metabolic pathways such as TOR signaling or IIS (see Figure 2), which upon inhibition lead to both prolonged lifespan and elevated LD synthesis. The situation in mammals and humans is more difficult to interpret due to markedly larger cell numbers, the enhanced diversity of differentiated cell types, and the complex interaction among diverse tissues. It is striking that LDs are concomitant to age-related disease. Concerning this, however, we want to question the still prevailing concept that LDs, by oversimplification understood as “monofunctional” fat-accumulating vesicles, are causative of the pathogenesis of age-related diseases. Taken together, the existing literature advocates a different view, suggesting that LDs represent multifunctional organelles of particular physiological relevance that play a subtle, *Janus-faced* role in disease: LDs essentially fulfilling a protective, retarding function during early pathogenetic stages, but converting to the opposite function in the course of disease progression when an excessive accumulation of LDs amplifies a phenotype characteristic of advanced disease states. 

Moreover, disregarding the pathogenic aspect, there is evidence for a physiological role of LDs as important “players” in healthy aging in humans. It is well accepted that the Mediterranean diet has numerous beneficial effects on human health. Many studies have shown that this diet reduces mortality and lowers the risk of developing cancer, neurodegenerative diseases, and cardiovascular diseases [493]. Some of the effects of the Mediterranean diet can be attributed to sirtuins [494], which have been addressed as regulators of LD biogenesis at several instances in this review. This puts emphasis on beneficial nutritional aspects, in particular focusing on two essential pillars of the Mediterranean diet: red wine and olive oil. In fact, it was shown that resveratrol, a polyphenol enriched in red wine, is an activator of sirtuin Sir2p (the yeast homologue of Sirt1) that has the capability to extend the lifespan in a broad variety of organisms [83]. It has to be noted critically that the activation of yeast Sirt1 by resveratrol occurs in an indirect mode via the cAMP-Epac1-AMPK-Sirt1 pathway, with Sirt1 being likely to be activated by increased cellular amounts of NAD^+^ [495]. Recently it was shown that monounsaturated fatty acids such as oleic acid, the main component of olive oil, allosterically activate Sirt1 at a magnitude many times higher than that of resveratrol [359]. Considering the connection between Sirt signaling and LD biogenesis, it would be thrilling to see, in the future, if some of the positive effects of the Mediterranean diet can be attributed to the stimulation of LD biogenesis. 

**Figure 3:**
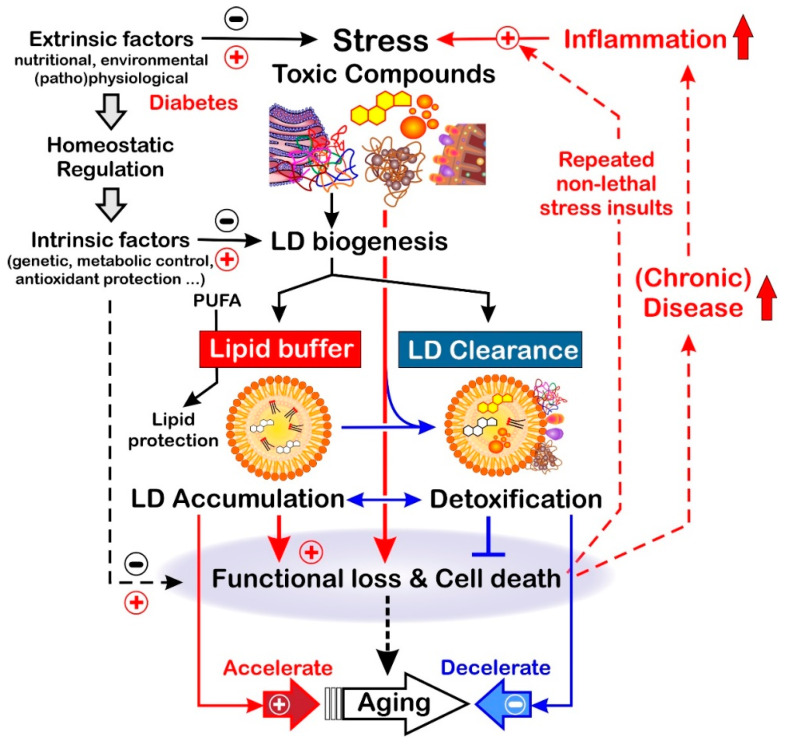
Model for the Janus-faced role of LDs in the aging process. Central to this explanatory approach is the bifunctional involvement of LDs in cellular maintenance, with LDs serving as both (i) a dynamic lipid /fat buffer, and (ii) a “sink” for toxic compounds upon LD clearance (proteins, lipids, and toxic compounds). In addition, LDs may also provide lipid protection by preserving PUFAs from excessive LPO and can quench stress derived from extrinsic factors. Under stress conditions, such as those created by ROS, the ER, or mitochondria, stimulation of LD biogenesis becomes the most important factor, and excessive LD formation depends on a variety of intrinsic factors (TOR, IIS, and TGF-β). The increase in the LD pool establishes a delicate balance between LD accumulation and LD-based detoxification. This balance determines the outcome of the stress response, which protects against cell death but may result in a chronic process (disease/inflammation) based on it. In contrast with the initial beneficial effects, the accumulation of LD accelerates the progression of chronic disease and thus the “aging” process. Hence, in stressed cells, LD biogenesis and LD functionality, both indirectly and directly, intervenes with multifaceted cellular life–death decisions (shaded blue area).

In synopsis, it is obvious that lipid metabolism is closely linked to aging and cellular stress responses via highly complex interactions that are not yet fully understood. According to our recent knowledge, it can be concluded that LDs participate in these complex metabolic, aging-associated networks by playing a “Janus-faced” role, as illustrated in Figure 3, and it will be the subject of future investigation to elucidate the exact, underlying contexts in detail.

## Figures and Tables

**Table 1:** Age-related diseases associated with an increase in cellular LD numbers.

Disease	Main Affected Cell Type/Tissue	References
Alzheimer’s disease	neurons, glia, myeloid cells, ependymal cells, astrocytes	[319,320,321,322,323]
Parkinson’s disease	neurons, microglia	[319,324,325]
Age-related macular degeneration	retinal pigment epithelium	[326]
Stroke	microglia	[327]
Atherosclerosis	Foam cells	[328,329]
Cardiovascular disease	myocardium	[330,331]
Sarcopenia	muscle cells	[332,333]
Rheumatoid arthritis	T-cells	[334]
Chronic obstructive pulmonary disease (COPD)	macrophages	[327]
Periodontitis	monocytes, macrophages	[335,336]
Osteopenia	osteoblasts, osteocytes	[337,338]
Osteoarthritis	chondrocytes, cartilage	[339,340,341]
Diabetes	β-cells	[342,343]
Liver disease (NAFLD) ^1^	parenchymal hepatocytes	[344] ^2^
Cancer	several	[343,345,346]
Senescence	several	[347,348,349]

^1^ Non-alcoholic fatty liver disease per se is characterized by progressive steatosis involving LD accumulation. ^2^ Review of the aging aspect of NAFLD to HCC progression.

## Data Availability

Not applicable.

## Abbreviations

AD, Alzheimer’s disease; AMPK, AMP-activated protein kinase; ANT, adenine-nucleotide translocator; Apo and APO, apolipoproteins; ARDs, age-related diseases; ATF6, activating transcription factor 6; ATG, autophagy related genes; ALS, amyotrophic lateral sclerosis; CBS, cystathionine β-synthase; Ces1d, cholesteryl-ester hydrolase; CCN1, central communication network factor 1; DFP, deferiprone; ECM, extracellular matrix; ER, endoplasmic reticulum; ESCRT, endosomal sorting complexes required for transport; Fe-S, iron-sulfur clusters; FOXO, forkhead box O; FRDA, Friedreich’s Ataxia; Ftx, frataxin; GAP, GTPase activating protein; GSH, glutathione (reduced from); GSSG, the oxidized (disulfide) form of GSH; HCC, hepatocellular carcinoma; HDACs, histone deacetylases; HNE, 4-hydroxy-2-nonenal; HNF1β, hepatocyte nuclear factor 1β; HSCs, hepatic stellate cells; IBs, inclusion bodies; IIS, Insulin/Insulin growth factor -1 signaling; ISC, intestinal stem cell; ISCU, iron–sulfur cluster forming unit; JNK, c-jun amino-terminal kinase; LDs, Lipid droplets; LDL, low-density lipoprotein; oxLDL, oxidized LDL; Lox, peroxidized lipids; LPO, lipid peroxidation; LROs, lysosome-related organelles; MAC, mitochondrial-apoptosis-induced channel; MAGIC, mitochondria as guardian in cytosol; MAT, marrow adipose tissue; MOMP, mitochondrial outer membrane permeabilization; MSCs, bone marrow stromal cells; mPT, mitochondrial permeability transition pore; NAFLD, non-alcoholic fatty liver disease; NASH, non-alcoholic steatohepatitis; nLDs, nuclear LDs; NOX1, NADPH oxidase 1; PA, phosphatic acid; PCD, programmed cell death; PD, Parkinson’s disease; PERK, PKR-like ER kinase; PLIN, perilipin; PML, promyelocytic leukemia protein; PPAR, peroxisome proliferator-activated receptor; PUFAs, polyunsaturated fatty acids; rad, radiation damage; *raptor*, regulatory associated protein of mTOR; RCT, reverse cholesterol transport; *rictor*, rapamycin-insensitive companion of mTOR; ROS, reactive oxygen species; SIRT, sirtuin; TAG, triacylglycerol(s); TE, transposable element; TNFα, tumor necrosis factor α; TNFR, TNFα-receptor; TOR, target of rapamycin; mTORC, mammalian TOR complex; TSC, trans-sulfuration pathway; UPR, unfolded protein response; UPR^ER^, ER stress-associated UPR; TSC, tuberous sclerosis complex; VDAC, voltage-dependent anion channel; VLDL, very-low-density lipoprotein; VSMCs, vascular smooth muscle cells; Xbp1, X-Box binding protein 1.
